# Design, Synthesis, and *In Vivo* Evaluation of a New Series of Indole-Chalcone Hybrids as Analgesic and Anti-Inflammatory Agents

**DOI:** 10.1021/acsomega.4c00026

**Published:** 2024-02-28

**Authors:** Iman Baramaki, Mehlika Dilek Altıntop, Rana Arslan, Feyza Alyu Altınok, Ahmet Özdemir, Ilhem Dallali, Ahmed Hasan, Nurcan Bektaş Türkmen

**Affiliations:** † Laboratory of Neurotherapeutics, Drug Research Program, Division of Pharmacology and Pharmacotherapy, Faculty of Pharmacy, University of Helsinki, 00014 Helsinki, Finland; ‡ Department of Pharmaceutical Chemistry, Faculty of Pharmacy, Anadolu University, 26470 Eskişehir, Turkey; § Department of Pharmacology, Faculty of Pharmacy, Anadolu University, 26470 Eskişehir, Turkey; ∥ Department of Pharmacology, Graduate School of Health Sciences, Anadolu University, 26470 Eskişehir, Turkey

## Abstract

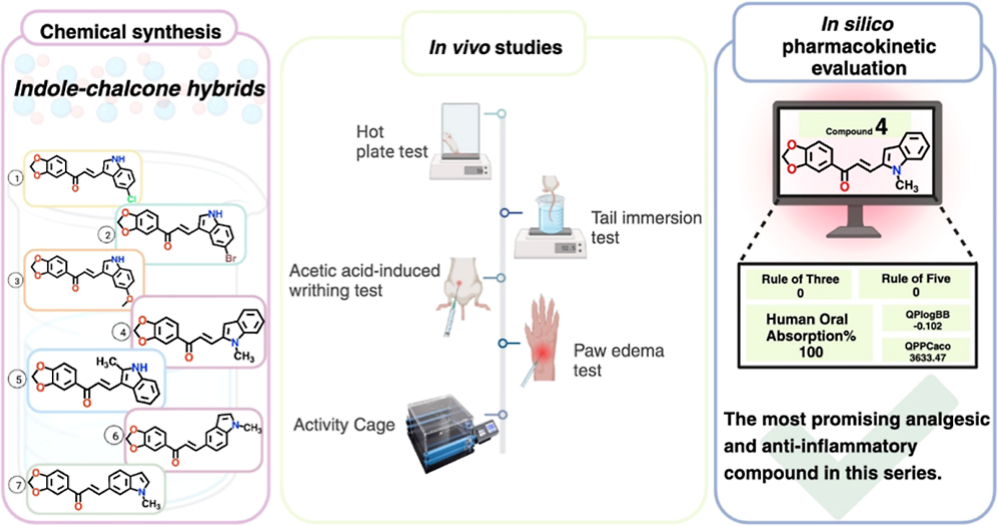

Indole-chalcone hybrids have burst into prominence as potent weapons in the battle against pain and inflammation due to their unique features, allowing these ligands to form pivotal interactions with biological targets. In this context, the base-catalyzed Claisen–Schmidt condensation of 3′,4′-(methylenedioxy)acetophenone with heteroaromatic aldehydes carrying an indole scaffold yielded new chalcones (**1**–**7**). The central and peripheral antinociceptive activities of all chalcones (compounds **1**–**7**) at the dose of 10 mg/kg (*i.p*.) were evaluated by hot plate (supraspinal response), tail immersion (spinal response), and acetic acid-induced writhing tests in mice. The anti-inflammatory activities of compounds **1**–**7** were also investigated by means of a carrageenan-induced mouse paw edema model. The results revealed that compounds **1**–**7** extended the latency of response to thermal stimulus significantly in a hot-plate test similar to dipyrone (300 mg/kg; *i.p*.), the positive control drug. However, only compounds **2**–**7** were found to be significantly effective in the tail-immersion test. Compounds **1**–**7** also significantly showed analgesic effect by reducing the number of writhes and anti-inflammatory activity by inhibiting edema formation at different time intervals and levels. 1-(1,3-Benzodioxol-5-yl)-3-(1-methyl-1*H*-indol-2-yl)prop-2-en-1-one (**4**) drew attention by providing the highest efficacy results in both acute analgesia and inflammation models. Based on the *in silico* data acquired from the QikProp module, compound **4** was predicted to possess favorable oral bioavailability and drug-like properties. Taken together, it can be concluded that chalcones (**1**–**7**), especially compound **4**, are outstanding candidates for further research to investigate their potential use in the management of pain and inflammation.

## 1 Introduction

Pain and inflammation, which encompass key pathophysiological processes implicated in various disease states, are intricately connected. Notably, inflammation underlies the perception of most pain sensations, including acute pain and other pain modalities.^1−3^ The complexity of pain perception and the underlying mechanisms at both the peripheral and central levels necessitate a diverse arsenal of drugs to combat these pathological conditions. To address the limitations associated with current anti-inflammatory and analgesic drugs, including nonsteroidal anti-inflammatory drugs (NSAIDs) with known side effects and tolerability concerns, novel drug research and development in this area remain imperative.^4,5^

Chalcones are considered open-chain flavonoids in which two aromatic rings are joined by a three-carbon α,β-unsaturated carbonyl system, which acts as a Michael acceptor group, allowing these ligands to effectively bind to various biological targets^6−13^ and therefore these compounds exhibit a diverse range of biological activities such as analgesic,^14−19^ anti-inflammatory,^16,20−22^ antidepressant,^15,16^ and anxiolytic^23,24^ activities. Consequently, natural and synthetic chalcones have captured the attention of researchers in the exploration of novel drug development.^6−13^ Additionally, chalcones have already demonstrated pharmacological significance in the pharmaceutical market.^7,25^ Several existing studies are further exemplifying the clinical potential of chalcones.^26,27^

**Figure 1:**
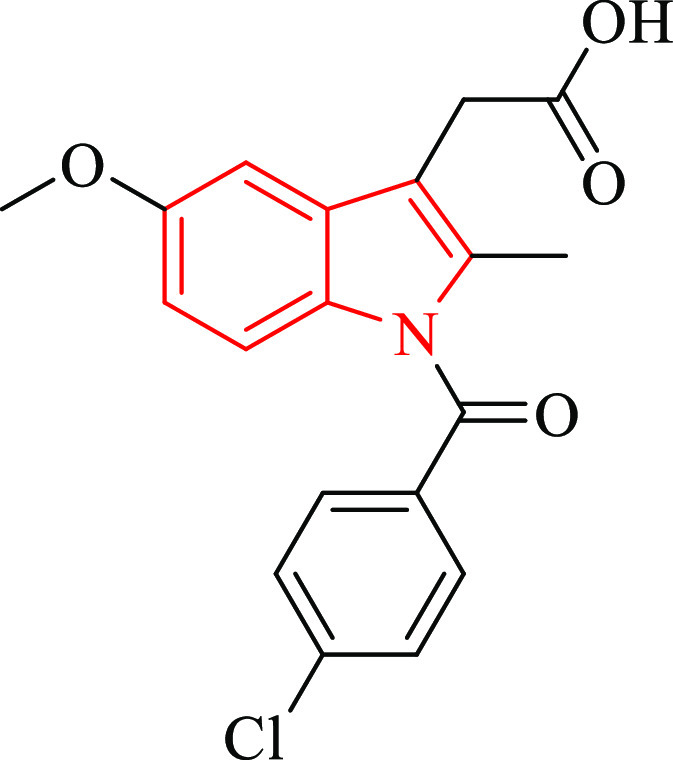
Indomethacin.

Indole is one of the top 25 most common nitrogen heterocycles in pharmaceuticals approved by the U.S. Food and Drug Administration (FDA). Indole is not only a vital component of endogenous substances in the body such as tryptophan (an essential amino acid) and serotonin (a monoamine neurotransmitter), but also an indispensable scaffold found in the structure of natural (e.g., vinca alkaloids) and synthetic drugs.^28,29^ In particular, the pharmacological applications of indole for the management of pain and inflammation make it one of the most eligible scaffolds for the discovery of analgesic and anti-inflammatory drugs. Indomethacin (Figure 1) ranks among the most commonly prescribed NSAIDs, exerting its action through the inhibition of cyclooxygenases (COXs).^30−33^

Based on the aforementioned data and our previous work on indole-chalcone hybrids exerting *in vivo* anti-inflammatory activity,^22^ herein, we synthesized new indole-chalcone hybrids (**1**–**7**) and evaluated their *in vivo* analgesic effects using hot plate, tail immersion, and acetic acid-induced writhing tests, as well as their *in vivo* anti-inflammatory activities using a carrageenan-induced paw edema model. This comparative investigation aims to elucidate the potential of these indole-chalcone hybrids and to offer promising avenues for future drug development and clinical applications.

## 2 Results

**Scheme 1:**
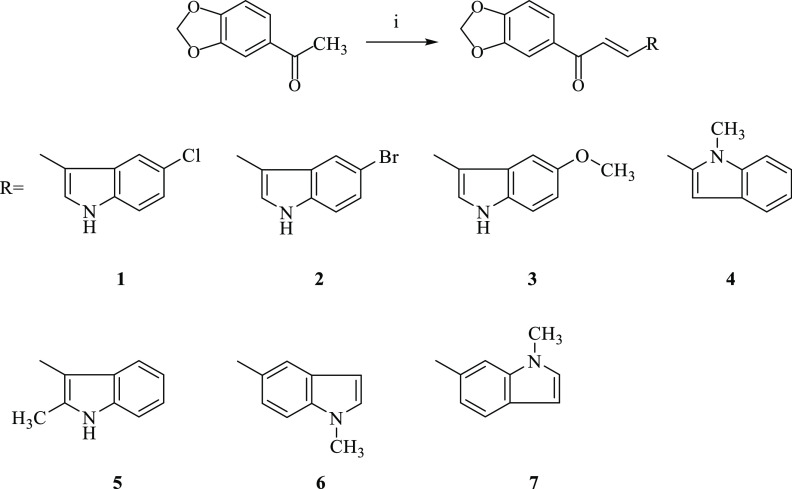
Synthetic Route for the Preparation of Indole-Chalcone Hybrids (**1**–**7**) Reagents and conditions: (i) R-CHO, 40% (w/v) NaOH, absolute ethanol, rt, 48 h.

The indole-chalcone hybrids (**1**–**7**) were obtained by means of the Claisen–Schmidt condensation of 3′,4′-(methylenedioxy)acetophenone with heteroaromatic aldehydes carrying an indole ring under basic conditions (Scheme 1). Their chemical structures were verified by infrared (IR), ^1^H nuclear magnetic resonance (NMR), and high-resolution mass spectrometry (HRMS) data.

**Figure 2:**
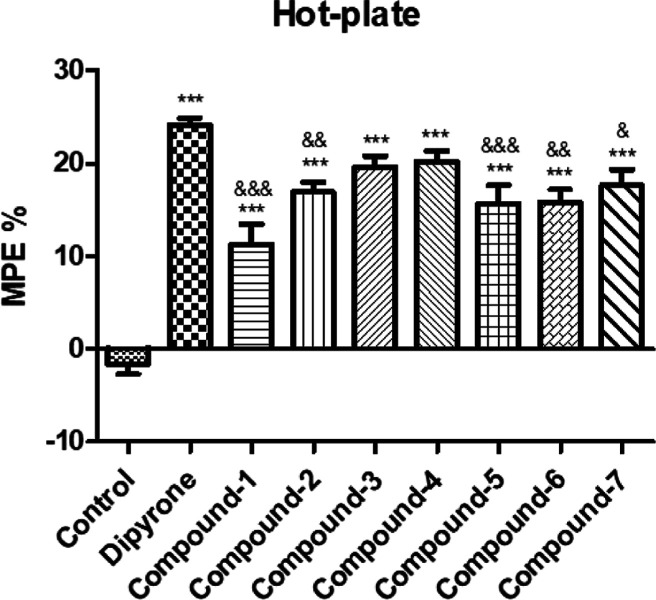
Antinociceptive effects of compounds **1**–**7** (10 mg/kg) and dipyrone (300 mg/kg) in the hot-plate test. *p < 0.05, **p < 0.01, and ***p < 0.001; significant difference compared to the control group. ^&^*p* < 0.05, ^&&^*p* < 0.01, ^&&&^*p* < 0.001; significant difference compared to the dipyrone group. Data are expressed as mean ± standard error (SE) (*n* = 8).

The impact of the compounds administered at a dose of 10 mg/kg on pain thresholds in the hot-plate test is illustrated in Figure 2. Compounds **1**–**7** significantly elevated pain thresholds compared to those of the control group and the positive control, dipyrone. In particular, only compounds **3** and **4** exhibited significant antinociceptive activity close to that of dipyrone.

**Figure 3:**
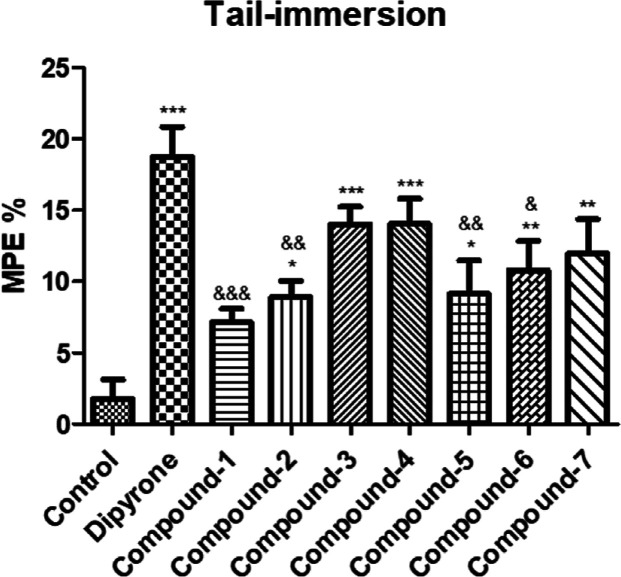
Antinociceptive effects of compounds **1**–**7** (10 mg/kg) and dipyrone (300 mg/kg) in the tail-immersion test. *p < 0.05, **p < 0.01, ***p < 0.001; significant difference compared to the control group. ^&^*p* < 0.05, ^&&^*p* < 0.01, ^&&&^*p* < 0.001; significant difference compared to the dipyrone group. Data are expressed as mean ± SE (*n* = 8).

The impact of the compounds administered at a dose of 10 mg/kg on pain thresholds in the tail-immersion test is illustrated in Figure 3. Except for compound **1**, compounds **2**–**7** significantly elevated pain thresholds compared to the control group, indicating their antinociceptive effects. Notably, only compounds **3** and **4** exhibited significant activity, like dipyrone.

**Figure 4:**
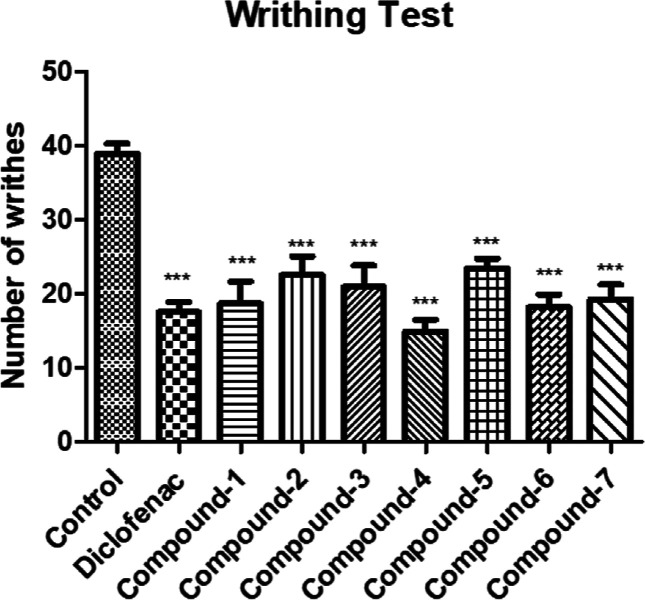
Antinociceptive effects of compounds **1**–**7** (10 mg/kg) and diclofenac potassium (30 mg/kg) in an acetic acid-induced writhing test. *p < 0.05, **p < 0.01, and ***p < 0.001; significant difference compared to the control group. Data are expressed as mean ± SE (*n* = 8).

In Figure 4, the impact of the compounds administered at a dose of 10 mg/kg on the recorded count of writhes in the acetic acid-induced writhing test is shown. Among the animals treated with compounds **1**–**7**, a statistically significant decrease in the number of writhing movements compared to that of the control group is observed. This decline is comparable to the decrease observed in the group treated with 30 mg/kg diclofenac potassium, which serves as the reference drug.

As indicated in Table 1, the administration of 10 mg/kg compounds **1**–**7** and 30 mg/kg diclofenac potassium caused a decrease in the number of acetic acid-induced writhing behaviors. The inhibition percentage of compound **4** (61.74%) was determined to be the highest.

**Table 1:** Inhibition % Caused by Compounds **1**–**7** (10 mg/kg) and Diclofenac Potassium (30 mg/kg) in the Acetic Acid-Induced Writhing Test

compound	inhibition %^a^
compound **1**	52.09
compound **2**	42.12
compound **3**	46.30
compound **4**	61.74
compound **5**	39.87
compound **6**	53.38
compound **7**	50.48
diclofenac potassium	54.98

a Inhibition % = [(number of writhes of the control group – number of writhes of the test group)/number of writhes of the control group] × 100.^34^

**Figure 5:**
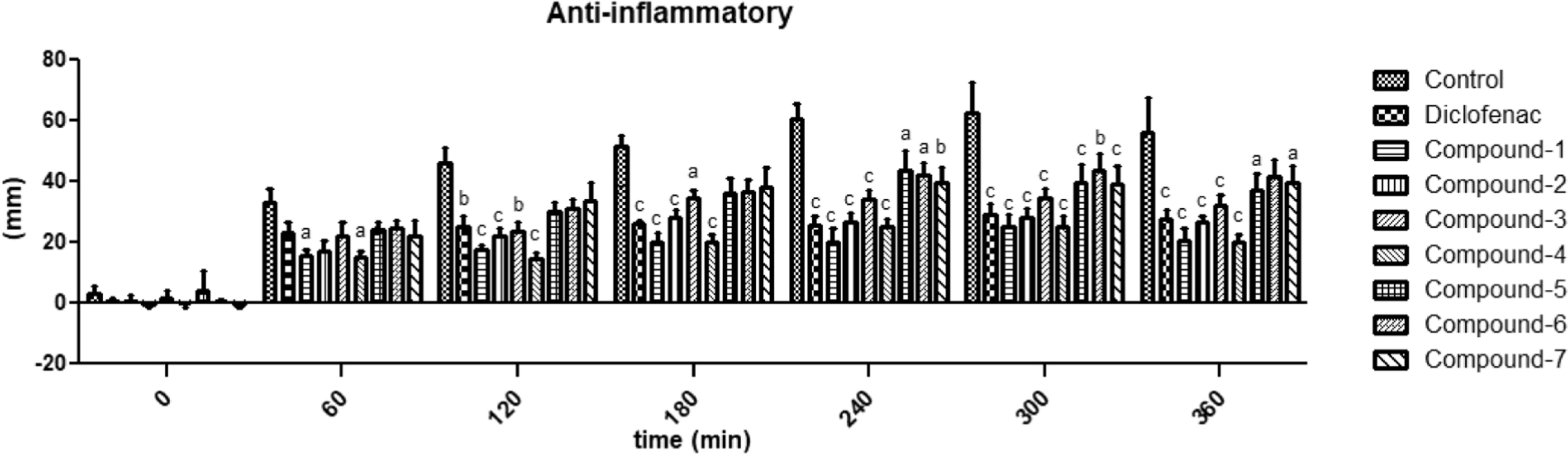
Anti-inflammatory effects of compounds **1**–**7** (10 mg/kg) and diclofenac potassium (30 mg/kg) in the carrageenan-induced paw edema test. ^a^*p* < 0.05, ^b^*p* < 0.01, and ^c^*p* < 0.001; significant difference compared to the control group. Data are expressed as mean ± SE (*n* = 8).

The anti-inflammatory activities of compounds **1**–**7** administered at a dose of 10 mg/kg are depicted in Figure 5 within the time intervals of 60–360 min. The significant effect of compound **1** in comparison to the control group commenced at 60 min and persisted for 360 min (at 60 min: ^a^*p* < 0.05, 120–360 min: ^c^*p* < 0.001). Compound **2** exhibited a significant effect compared to the control group, starting at 120 min and continuing for 360 min (at 120–360 min: ^c^*p* < 0.001). The significant effect of compound **3** compared to the control group started at 120 min and continued for 360 min (at 120 min: ^b^*p* < 0.01, 180 min: ^a^*p* < 0.05, 240–360 min: ^c^*p* < 0.001). Compound **4** showed a significant effect compared to the control group, beginning at 60 min and lasting for 360 min (at 60 min: ^a^*p* < 0.05, 120–360 min: ^c^*p* < 0.001). Compound **5** exhibited a significant effect in the time interval of 240–360 min compared to the control group (at 240 min: ^a^*p* < 0.05, 300 min: ^c^*p* < 0.001, 360 min: ^a^*p* < 0.05). Compound **6** only displayed significant effects at 240 and 300 min, with its effect concluding at 360 min (at 240 min: ^a^*p* < 0.05, 300 min: ^b^*p* < 0.01). The effect of compound **7** was significant in the time interval of 240–360 min compared to the control group (at 240 min: ^b^*p* < 0.01, 300 min: ^c^*p* < 0.001, 360 min: ^a^*p* < 0.05). The significant activity of the positive control drug diclofenac potassium began at 120 min and continued for 360 min (at 120 min: ^b^*p* < 0.01, 180–360 min: ^c^*p* < 0.001).

**Figure 6:**
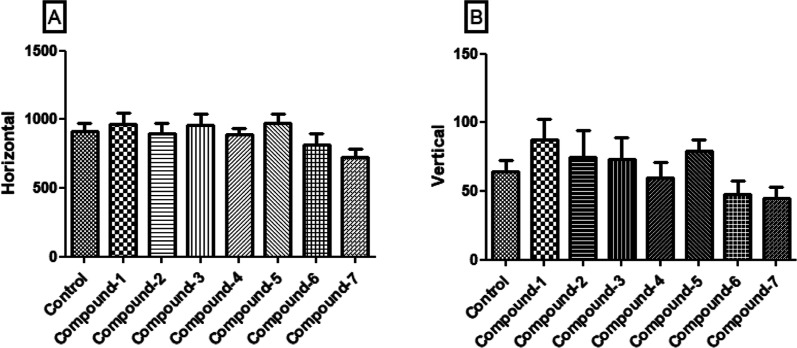
Effects of compounds **1**–**7** (10 mg/kg) on the horizontal (A) and vertical (B) movements in the activity cage. Data are expressed as mean ± SE (*n* = 8).

The horizontal (A) and vertical (B) movements of the mice within the activity cage are depicted in Figure 6. While there were slight variations observed in the vertical movements following the administration of compounds **1**–**7** (10 mg/kg), no significant alterations were noted in either of these parameters compared to the control group.

## 3 Discussion

### 3.1 *In Vivo* Studies

In this study, the potential antinociceptive and anti-inflammatory properties of new indole-chalcone hybrids (**1**–**7**) were explored.

Acute pain, characterized by rapid onset and short duration, often arises from tissue damage or injury (International Association for the Study of Pain (IASP) 2020, retrieved from https://www.iasp-pain.org). The development of acute pain involves inflammation, where inflammatory mediators like prostaglandins sensitize nociceptors, contributing to pain perception.^1^ Clinically used NSAIDs, such as ibuprofen and diclofenac potassium, not only inhibit COXs to exert anti-inflammatory effects but also modify nociceptor sensitivity, making them effective in pain management.^35^ Moreover, well-established central mechanisms, including the descending inhibitory pathway, play a role in the activities of some NSAIDs, such as indomethacin.^36^ Medications employing multiple mechanisms in clinical practice offer advantages for effectively addressing pain or inflammation. In this research, seven chalcone-based compounds were synthesized, and their efficacy was evaluated in the hot-plate (supraspinal) and tail-immersion (spinal) tests, standard for central analgesic responses.^37−39^

In the hot-plate test, all compounds showed effects like dipyrone. Notably, the effects of compounds **3** and **4** were close to that of dipyrone, indicating their involvement in central pathways (Figure 2). It was hypothesized that the compounds could exert analgesia through supraspinal action, as suggested by their observed responses in the hot plate test. In the tail-immersion test, all compounds except compound **1** exhibited varying levels of pharmacological effects. In this test, compounds **3** and **4** showed similar effects to dipyrone. The compounds that exerted activity in the tail immersion test were speculated to induce analgesia by influencing pain signaling at the spinal level (Figure 3). For the assessment of acute pain effects, the alternate preferred method is an acetic-acid-induced writhing test. The writhing test is a chemical method used to induce peripherally originated pain in mice through the injection of irritants such as acetic acid. Compounds that are effective through both central and peripheral mechanisms show activity in the writhing test.^40^ In this test as well, all chalcones exhibited pharmacological effects, similar to diclofenac potassium, the reference drug (Figure 4). These results imply that both central and peripheral processes are involved in the efficacy of compounds **1**–**7** in diminishing acute pain sensation in an acetic-acid-induced writhing test. The injection of acetic acid can trigger the release of a range of chemicals, including prostaglandins, which contribute to the sensitization of nociceptors and the induction of pain.^41^ This implies that chalcones might interact with pathways, altering nociceptor sensitivity. Remarkably, compound **4**, which exhibited the highest activity in methodologies assessing central activity, showed greater maximum inhibition (61.74%) than diclofenac potassium (54.98%) in the test assessing more peripheral activity (Table 1).

In the next stage of the study, the carrageenan-induced paw edema test was performed to investigate the anti-inflammatory properties of the chalcones. The carrageenan-induced paw edema assay is a widely used experimental model to evaluate acute inflammation.^42−44^ Carrageenan, a sulfated polysaccharide derived from red seaweed, is administered to animals, leading to localized tissue inflammation. This model mimics the early stages of inflammation and involves the release of inflammatory mediators such as prostaglandins and cytokines, resulting in increased vascular permeability and edema formation. The severity of paw swelling serves as an indicator of the anti-inflammatory potential of test compounds.^45,46^ In this assay, all tested compounds exerted anti-inflammatory action by inhibiting edema formation at different time intervals and levels. Compounds **1** and **4** exhibited the earliest onset of effects, while compounds **5** and **6** exhibited a later onset. Notably, compound **4** was found to be relatively the most effective agent. Conversely, the effects of compounds **5** and **6** were weaker and shorter. Despite these variations, it can be broadly concluded that all compounds exhibit anti-inflammatory effects, like diclofenac potassium, albeit with varying degrees of potency (Figure 5).

It is essential to find out the side effect profiles of medications and correlate them with their pharmacological properties, especially for those that show analgesic effects through central mechanisms. In this regard, assessing whether the administration of the test compounds affects motor functions becomes imperative. Thus, the experiment employed in this study involved the use of an activity cage, which is a frequently utilized method for quantifying motor activity. Apart from changes, especially in vertical movements, no statistically significant effects were observed. However, these alterations were considered insignificant within the scope of the administered dose and the prevailing experimental conditions (Figure 6). Upon a comprehensive evaluation of the collective findings, it becomes evident that the synthesized chalcones showcase both antinociceptive and varying degrees of anti-inflammatory effects. Importantly, these effects are not influenced by alterations in locomotor activity.

Indole-chalcone hybrids (**1**–**7**) exhibit the potential to exert their actions through central or peripheral mechanisms, leading to antinociceptive outcomes and displaying diverse anti-inflammatory effects over varying levels and durations. This collective data underscores the promising prospect of these compounds as potential candidates for drug development. Their ability to elicit effects through multiple mechanisms could offer clinical advantages. However, further pharmacological studies should be conducted to thoroughly understand these effects and their possible mechanisms of action.

### 3.2 *In Silico* Pharmacokinetic Evaluation

*In silico* approaches for predicting of the pharmacokinetic profiles of drug candidates occupy a prominent place in the early stages of drug discovery to save time and money and avoid the ethical problems arising from the large number of animals required for *in vivo* experiments.^47,48^ Compounds **1**–**7** were evaluated for their *in silico* pharmacokinetic profiles by running QikProp, a predictive ADME module within the Maestro suite produced by Schrödinger (Table 2). The CIQPlogS (ranging from −6.351 to −4.948) and QPlogPo/w (ranging from 3.514 to 4.016) values of compounds **1**–**7** were within the range suggested by QikProp.

**Table 2:** Predicted Pharmacokinetic Features of Compounds **1**–**7**

property or descriptor	compounds						
	**1**	**2**	**3**	**4**	**5**	**6**	**7**
#stars^a^	1	1	0	1	0	1	1
SASA^a^	550.423	555.464	562.756	556.539	544.084	560.778	560.767
CIQPlogS^a^	–5.439	–6.351	–5.058	–4.948	–5.023	–4.948	–4.948
QPlogPo/w^a^	3.913	3.990	3.514	3.992	3.709	4.016	4.016
QPPCaco^a^	2000.708	2000.703	2000.027	3633.470	2306.835	3641.325	3641.106
QPlogBB^a^	–0.180	–0.169	–0.417	–0.102	–0.279	–0.104	–0.104
QPPMDCK^a^	2580.268	2774.474	1046.488	1995.223	1221.034	1999.885	1999.755
QPlogKhsa^a^	0.361	0.384	0.257	0.316	0.370	0.333	0.333
human oral absorption %^a^	100.000	100.000	100.000	100.000	100.000	100.000	100.000
rule of five^b^	0	0	0	0	0	0	0
rule of three^b^	0	0	0	0	0	0	0

a #stars: number of property or descriptor values that fall outside the 95% range of similar values for known drugs (recommended range: 0–5). SASA: total solvent accessible surface area in square angstroms using a probe with a 1.4 Å radius (recommended range: 300.0–1000.0). CIQPlogS: conformation-independent predicted aqueous solubility (recommended range: −6.5 to 0.5). QPlogPo/w: predicted octanol/water partition coefficient (recommended range: −2.0 to 6.5). QPPCaco: predicted apparent Caco-2 cell permeability in nm/s (<25 poor, >500 great). QPlogBB: predicted brain/blood partition coefficient (recommended range: −3.0 to 1.2). QPPMDCK: predicted apparent MDCK cell permeability in nm/s (<25 poor, >500 great). QPlogKhsa: prediction of binding to human serum albumin (recommended range: −1.5 to 1.5). Human oral absorption %: predicted human oral absorption on a 0 to 100% scale (>80% is high, <25% is poor).

b Rule of five: number of violations of Lipinski’s rule of five. The rules are molecular weight of the molecule <500, QPlogPo/w < 5, hydrogen bond donor atoms ≤5, hydrogen bond acceptor atoms ≤10. Compounds that provide these rules are considered drug-like molecules (maximum is 4). Rule of three: number of violations of Jorgensen’s rule of three. The three rules are predicted aqueous solubility (QPlogS) > −5.7, predicted apparent Caco-2 cell permeability (QPPCaco in nm/s) > 22 nm/s, #primary metabolites <7 (maximum is 3). Compounds with fewer (and preferably no) violations of these rules are more likely to be orally available agents (Schrödinger Release 2022-2, LLC, New York, USA).

Oral administration is the most preferred route for drugs due to its ease of administration and patient compliance. The Caco-2 cell monolayer model is used as a common surrogate for predicting the *in vitro* human intestinal permeability of a drug candidate owing to its morphological and functional similarity with human enterocytes.^49^ The QPPCaco values of compounds **1**–**7**, which were found to be >500, pointed out the potential for intestinal permeability of these compounds.

Based on *in silico* data, the drug-likeness parameter (indicated by #stars) for all compounds was found to be within the optimum range. All compounds are estimated to possess good oral absorption (100%). As indicated in Table 2, none of the compounds violated Lipinski’s and Jorgensen’s parameters, making them potential orally bioavailable analgesic and anti-inflammatory agents endowed with favorable drug-like properties.

Delivery of many therapeutic agents into the central nervous system (CNS) is restricted by the blood–brain barrier (BBB), which remains a significant bottleneck for the development of new CNS-targeted drugs.^50^ For this purpose, the prediction of the brain/blood partition coefficient, QPlogBB, was performed by QikProp for each compound. The QPlogBB values of all compounds were found to be within the recommended range. Predicted apparent Madin–Darby canine kidney (MDCK) cell permeability (in nm/s) is also a crucial parameter since MDCK cells are considered to be a good mimic for the BBB.^50,51^ Their QPPMDCK values were also within the designated range. Taken together, compounds **1**–**7** possess the probability of being able to successfully penetrate the BBB.

## 4 Conclusions

In this paper, the synthesis of new indole-chalcone hybrids (**1**–**7**) was performed efficiently. Hot plate, tail immersion, and acetic acid-induced writhing tests were employed to investigate their central and peripheral antinociceptive activities at a dose of 10 mg/kg (*i.p*.) in mice. Compounds **1**–**7** were also investigated for their anti-inflammatory activities using a carrageenan-induced mouse paw edema model. According to the data obtained by the hot-plate test, compounds **1**–**7** significantly prolonged the latency of response to thermal stimuli in a manner similar to dipyrone (300 mg/kg; *i.p*.). However, only compounds **2**–**7** were significantly effective in the tail-immersion test. Compounds **1**–**7** also significantly exerted analgesic action by diminishing the number of writhes in mice and anti-inflammatory action by preventing edema formation at different time intervals and levels. Considering the data collected from *in vivo* assays, in particular, compound **4** stands out as a promising candidate for further research to assess its potential use in the management of pain and inflammation. Based on *in silico* ADME evaluation, compound **4** is estimated to possess favorable oral bioavailability and drug-likeness.

## 5 Materials and Methods

### 5.1 Chemistry

All reagents were procured from commercial suppliers and used without further purification. Melting points (mp) were determined using an Electrothermal IA9200 digital melting point apparatus (Staffordshire, UK) and uncorrected. IR spectra were acquired from an IRPrestige-21 Fourier Transform Infrared spectrophotometer (Shimadzu, Tokyo, Japan). NMR spectra were recorded by employing a Bruker 300 MHz spectrometer (Bruker, Billerica, MA, USA). HRMS spectra were recorded on a Shimadzu LCMS-IT-TOF system (Shimadzu, Kyoto, Japan).

#### 5.1.1 Synthesis of 1-(1,3-Benzodioxol-5-yl)-3-(aryl)prop-2-en-1-one Derivatives (**1**–**7**)

3′,4′-(Methylenedioxy)acetophenone (0.005 mol) was reacted with heteroaromatic aldehyde (0.005 mol) in the presence of 40% aqueous NaOH (w/v) (5 mL) in absolute ethanol (30 mL) at room temperature (rt) for 48 h. Upon completion of the reaction, the reaction mixture was poured on crushed ice. The precipitate was filtered and washed with water. After drying, the product was crystallized from ethanol.^52^

##### 5.1.1.1 1-(1,3-Benzodioxol-5-yl)-3-(5-chloro-1*H*-indol-3-yl)prop-2-en-1-one (**1**)

Yield: 55%. mp: 195–196 °C. IR ν_max_ (cm^–1^): 3209.55, 3190.26, 3109.25, 3053.32, 2951.09, 2906.73, 2846.93, 1643.35, 1622.13, 1575.84, 1523.76, 1485.19, 1438.90, 1402.25, 1352.10, 1319.31, 1288.45, 1228.66, 1143.79, 1130.29, 1101.35, 1049.28, 891.11, 850.61, 785.03, 748.38, 732.95, 677.01, 611.43. ^1^H NMR (300 MHz, DMSO-*d*_6_): δ (ppm) 6.13 (s, 2H), 7.27–7.30 (m, 3H), 7.53–7.55 (m, 4H), 8.06–8.37 (m, 2H), 12.30 (brs, 1H). HRMS (ESI) (*m*/*z*): [M + H]^+^ calcd for C_18_H_12_ClNO_3_, 326.0578; found, 326.0584.

##### 5.1.1.2 1-(1,3-Benzodioxol-5-yl)-3-(5-bromo-1*H*-indol-3-yl)prop-2-en-1-one (**2**)

Yield: 54%. mp: 191–192 °C. IR ν_max_ (cm^–1^): 3217.27, 3103.46, 3053.32, 2949.16, 2902.87, 2841.15, 1639.49, 1620.21, 1573.91, 1523.76, 1427.32, 1392.61, 1346.31, 1319.31, 1288.45, 1228.66, 1126.43, 1095.57, 1041.56, 885.33, 848.68, 798.53, 781.17, 725.23, 671.23, 607.58. ^1^H NMR (300 MHz, DMSO-*d*_6_): δ (ppm) 6.13 (s, 2H), 7.38–7.42 (m, 3H), 7.48–7.51 (m, 4H), 8.21–8.36 (m, 2H), 12.31 (brs, 1H). HRMS (ESI) (*m*/*z*): [M + H]^+^ calcd for C_18_H_12_BrNO_3_, 370.0073; found, 370.0061.

##### 5.1.1.3 1-(1,3-Benzodioxol-5-yl)-3-(5-methoxy-1*H*-indol-3-yl)prop-2-en-1-one (**3**)

Yield: 31%. mp: 110–111 °C. IR ν_max_ (cm^–1^): 3163.26, 3107.32, 3055.24, 2951.09, 2904.80, 2816.07, 1639.49, 1622.13, 1602.85, 1585.49, 1523.76, 1485.19, 1435.04, 1390.68, 1361.74, 1286.52, 1259.52, 1209.37, 1180.44, 1138.00, 1107.14, 1070.49, 1033.85, 1024.20, 925.83, 839.03, 788.89, 713.66, 632.65. ^1^H NMR (300 MHz, DMSO-*d*_6_): δ (ppm) 3.78 (s, 3H), 6.13 (s, 2H), 6.88 (dd, *J* = 2.58 Hz, 8.82 Hz, 2H), 7.40 (d, *J* = 8.82 Hz, 3H), 7.57–7.58 (m, 3H), 8.21–8.22 (m, 1H), 12.03 (brs, 1H). HRMS (ESI) (*m*/*z*): [M + H]^+^ calcd for C_19_H_15_NO_4_, 322.1074; found, 322.1072.

##### 5.1.1.4 1-(1,3-Benzodioxol-5-yl)-3-(1-methyl-1*H*-indol-2-yl)prop-2-en-1-one (**4**)

Yield: 68%. mp: 128–129 °C. IR ν_max_ (cm^–1^): 3103.46, 3082.25, 3049.46, 2941.44, 2897.08, 1651.07, 1600.92, 1581.63, 1568.13, 1519.91, 1498.69, 1485.19, 1462.04, 1440.83, 1392.61, 1361.74, 1344.38, 1321.24, 1288.45, 1244.09, 1230.58, 1184.29, 1153.43, 1124.50, 1109.07, 1035.77, 1018.41, 968.27, 941.26, 921.97, 904.61, 879.54, 846.75, 817.82, 800.46, 752.24, 731.02, 680.87, 636.51, 624.94. ^1^H NMR (300 MHz, DMSO-*d*_6_): δ (ppm) 3.90 (s, 3H), 6.18 (s, 2H), 7.07–7.12 (m, 3H), 7.45–7.62 (m, 4H), 7.67–7.68 (m, 1H), 7.86–7.95 (m, 2H). HRMS (ESI) (*m*/*z*): [M + H]^+^ calcd for C_19_H_15_NO_3_, 306.1125; found, 306.1136.

##### 5.1.1.5 1-(1,3-Benzodioxol-5-yl)-3-(2-methyl-1*H*-indol-3-yl)prop-2-en-1-one (**5**)

Yield: 30%. mp: 199–200 °C. IR ν_max_ (cm^–1^): 3244.27, 3192.19, 3115.04, 3055.24, 2920.23, 2808.36, 1629.85, 1618.28, 1581.63, 1496.76, 1462.04, 1377.17, 1359.82, 1311.59, 1282.66, 1242.16, 1161.15, 1101.35, 1035.77, 960.55, 929.69, 867.97, 734.88, 702.09, 655.80, 621.08. ^1^H NMR (300 MHz, DMSO-*d*_6_): δ (ppm) 2.68 (s, 3H), 6.13 (s, 2H), 7.12–7.20 (m, 4H), 7.36–7.40 (m, 3H), 8.02–8.05 (m, 2H), 11.99 (brs, 1H). HRMS (ESI) (*m*/*z*): [M + H]^+^ calcd for C_19_H_15_NO_3_, 306.1125; found, 306.1135.

##### 5.1.1.6 1-(1,3-Benzodioxol-5-yl)-3-(1-methyl-1*H*-indol-5-yl)prop-2-en-1-one (**6**)

Yield: 75%. mp: 118–119 °C. IR ν_max_ (cm^–1^): 3103.46, 3082.25, 2941.44, 2895.15, 1643.35, 1602.85, 1583.56, 1566.20, 1504.48, 1485.19, 1438.90, 1382.96, 1359.82, 1294.24, 1242.16, 1149.57, 1130.29, 1109.07, 1097.50, 1033.85, 1020.34, 983.70, 972.12, 933.55, 914.26, 893.04, 846.75, 798.53, 763.81, 731.02, 719.45, 707.88, 642.30, 615.29. ^1^H NMR (300 MHz, DMSO-*d*_6_): δ (ppm) 3.83 (s, 3H), 6.12 and 6.16 (2s, 2H), 6.40–6.63 (m, 1H), 6.98–7.07 (m, 1H), 7.44–7.45 (m, 2H), 7.52 (d, *J* = 8.67 Hz, 1H), 7.62 (d, *J* = 8.19 Hz, 1H), 7.68–7.69 (m, 1H), 7.76–7.79 (m, 1H), 7.86–7.89 (m, 1H), 8.04–8.05 (m, 1H). HRMS (ESI) (*m*/*z*): [M + H]^+^ calcd for C_19_H_15_NO_3_, 306.1125; found, 306.1137.

##### 5.1.1.7 1-(1,3-Benzodioxol-5-yl)-3-(1-methyl-1*H*-indol-6-yl)prop-2-en-1-one (**7**)

Yield: 81%. mp: 142–143 °C. IR ν_max_ (cm^–1^): 3107.32, 3086.11, 2904.80, 2827.64, 1647.21, 1600.92, 1577.77, 1502.55, 1487.12, 1475.54, 1440.83, 1423.47, 1336.67, 1317.38, 1294.24, 1246.02, 1190.08, 1112.93, 1089.78, 1039.63, 1018.41, 979.84, 935.48, 912.33, 891.11, 844.82, 806.25, 769.60, 744.52, 719.45, 707.88, 648.08, 611.43. ^1^H NMR (300 MHz, DMSO-*d*_6_): δ (ppm) 3.87 (s, 3H), 6.12 and 6.17 (2s, 2H), 6.37–6.67 (m, 1H), 7.10 (d, *J* = 8.16 Hz, 2H), 7.47–7.48 (m, 1H), 7.59 (s, 2H), 7.68–7.69 (m, 1H), 7.87–7.91 (m, 2H), 8.02 (s, 1H). HRMS (ESI) (*m*/*z*): [M + H]^+^ calcd for C_19_H_15_NO_3_, 306.1125; found, 306.1131.

### 5.2 Pharmacology

#### 5.2.1 Animals

Balb/c male mice of 30–35 g were used. The animals were housed in well-ventilated rooms at 22 ± 1 °C with a 12 h light/dark cycle and had free access to standard pellets and water ad libitum. The animals were taken to the experimental room a few days before the experiments and were accustomed to the environment. Animals were habituated to the experimenter and apparatuses. Animals were randomly assigned to treatment groups, and the experiments were performed blind to avoid bias. Animal care and research protocols were performed strictly in accordance with Directive 2010/63/EU of the European Parliament and The Council and approved (no. 2021-31) by the Local Ethics Committee of Anadolu University, Eskişehir, Turkey.

#### 5.2.2 Experimental Groups

Distinct sets of experiments were established for assessments of all pharmacologic effects. Within each experimental set, a group of mice was injected with a solution containing 10% dimethyl sulfoxide (DMSO) in distilled water and served as the control group. The intraperitoneal (*i.p*.) administration of each chalcone derivative was performed at a dose of 10 mg/kg. The dose was determined based on the previous studies^15,19^ on analgesic and anti-inflammatory effects of chalcones. In the hot-plate and tail-immersion tests, 300 mg/kg of dipyrone (Sigma-Aldrich, St. Louis, MO, USA), *i.p*., was used as the reference drug. For the writhing and paw edema tests, 30 mg/kg of diclofenac potassium (Sigma-Aldrich, St. Louis, MO, USA), *i.p*., was utilized as the reference drug.

#### 5.2.3 Hot Plate Test

In this experimental procedure, a Ugo Basile (no. 7280) hot-plate set at 56 °C and enclosed by a plexiglass cylinder was employed. The time elapsed from when the animal was placed on the hot-plate until it exhibited one of the following responses: withdrawal of hind limbs, licking, elevation of hind limbs, or jumping, was measured as the response latency to the noxious stimulus^53^ and recorded. Measurements were taken both prior to and 30 min after substance administration. To prevent harm to the animals’ paws from the heat, a cutoff time of 20 s was established as the termination point for the experiment.

#### 5.2.4 Tail Immersion Test

A segment of the tail, measuring 3 cm from the tip, was immersed in water maintained at a temperature of 52.5 ± 0.2 °C within a beaker. The time from when the animal’s tail was submerged into the water until it was rapidly withdrawn from the water was measured and recorded as the response latency to the noxious stimulus.^54^ Measurements were taken both prior to and 30 min after substance administration. To prevent harm to the animals’ tails from the heat, a cutoff time of 15 s was established as the termination point for the experiment. The effect of compounds on pain thresholds was presented as the percentage of the maximal possible effect (MPE %)^34^
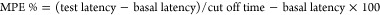


#### 5.2.5 Acetic Acid-Induced Writhing Test

Following a 45 min interval postadministration, the animals were injected with 0.6% (v/v) acetic acid (Sigma-Aldrich, St. Louis, MO, USA), *i.p*. After the 5 min waiting period, abdominal constrictions were then quantified for a period of 10 min and recorded as pain behavior.^55^ Abdominal constriction is characterized by extension of hind limbs backward, and dragging of the abdomen on the floor.^40^

#### 5.2.6 Paw Edema Test

Thirty minutes following *i.p*. injection of the test substance, 40 μL of 1% λ-carrageenan (Sigma-Aldrich, St. Louis, MO, USA) solution prepared in physiological saline was injected into each mouse’s right hind paw plantar tissue. As a control, 40 μL of physiological saline was injected into the left hind paw of the same animal. After inducing inflammation, the thickness resulting from paw edema was measured every 90 min over a 6 h period using a digital caliper.^46^

#### 5.2.7 Activity Cage

To assess spontaneous locomotor activity, the activity cage apparatus in the form of a plexiglass cage (Ugo Basile 47420, Varese, Italy) was utilized. This device emits IR beams along the opposite vertical edges, whereby the animal’s horizontal and vertical movements disrupt these beams, which are subsequently recorded through photosensors. The electronic system of the apparatus records and prints the measured data at predetermined intervals.^56^ After a 30 min interval following *i.p*. injection of vehicle or test compounds, each mouse was introduced into the activity cage for a duration of 15 min. During this time, both horizontal and vertical movements were monitored and recorded.

#### 5.2.8 Statistical Analysis

Statistical analysis was conducted using the GraphPad Prism version 5.0 software package. For the assessment of anti-inflammatory activity, two-way analysis of variance (ANOVA) followed by the Bonferroni test was employed. Additionally, for the evaluation of antinociceptive and locomotor activities, one-way analysis of variance (ANOVA) followed by Dunnett’s posthoc test was utilized. All results were presented as mean ± standard error (SE), and significance was determined at a threshold of *p* < 0.05.

### 5.3 Determination of *In Silico* Pharmacokinetic Profiles

QikProp, the *in silico* module within the Maestro suite produced by Schrödinger, was used to estimate crucial theoretical properties or descriptors of compounds **1**–**7** for the assessment of their absorption, distribution, metabolism, and elimination (ADME) profiles.

## Supporting Information Available

IR, ^1^H NMR, and HRMS spectra of compounds **1**–**7** (PDF)
