# Supplementary material for: Design, Synthesis, and In Vivo Evaluation of a New Series of Indole-Chalcone Hybrids as Analgesic and Anti-Inflammatory Agents
Source: ACS Omega. 2024 Feb 28;9(10):12175–83. doi: 10.1021/acsomega.4c00026 (PMC10938421; doi:10.1021/acsomega.4c00026)
Supplement: Supplementary file 1 — ao4c00026_si_001.pdf [file ao4c00026_si_001.pdf]

Supporting Information

Design, synthesis, and *in vivo* evaluation of a new series of indole-chalcone hybrids as analgesic and anti-inflammatory agents

Iman Baramaki[1], Mehlika Dilek Altıntop[2*], Rana Arslan[3], Feyza Alyu Altınok[3], Ahmet Özdemir[2], Ilhem Dallali[4], Ahmed Hasan[4], Nurcan Bektaş Türkmen[3]

[1] *Laboratory of Neurotherapeutics, Drug Research Program, Division of Pharmacology and Pharmacotherapy, Faculty of Pharmacy, University of Helsinki, 00014 Helsinki, Finland*

[2] *Department of Pharmaceutical Chemistry, Faculty of Pharmacy, Anadolu University, 26470 Eskişehir, Turkey*

[3] *Department of Pharmacology, Faculty of Pharmacy, Anadolu University, 26470 Eskişehir, Turkey*

[4] *Department of Pharmacology, Graduate School of Health Sciences, Anadolu University, 26470 Eskişehir, Turkey*

* Corresponding author.

Figure S1. IR spectrum of compound **1**

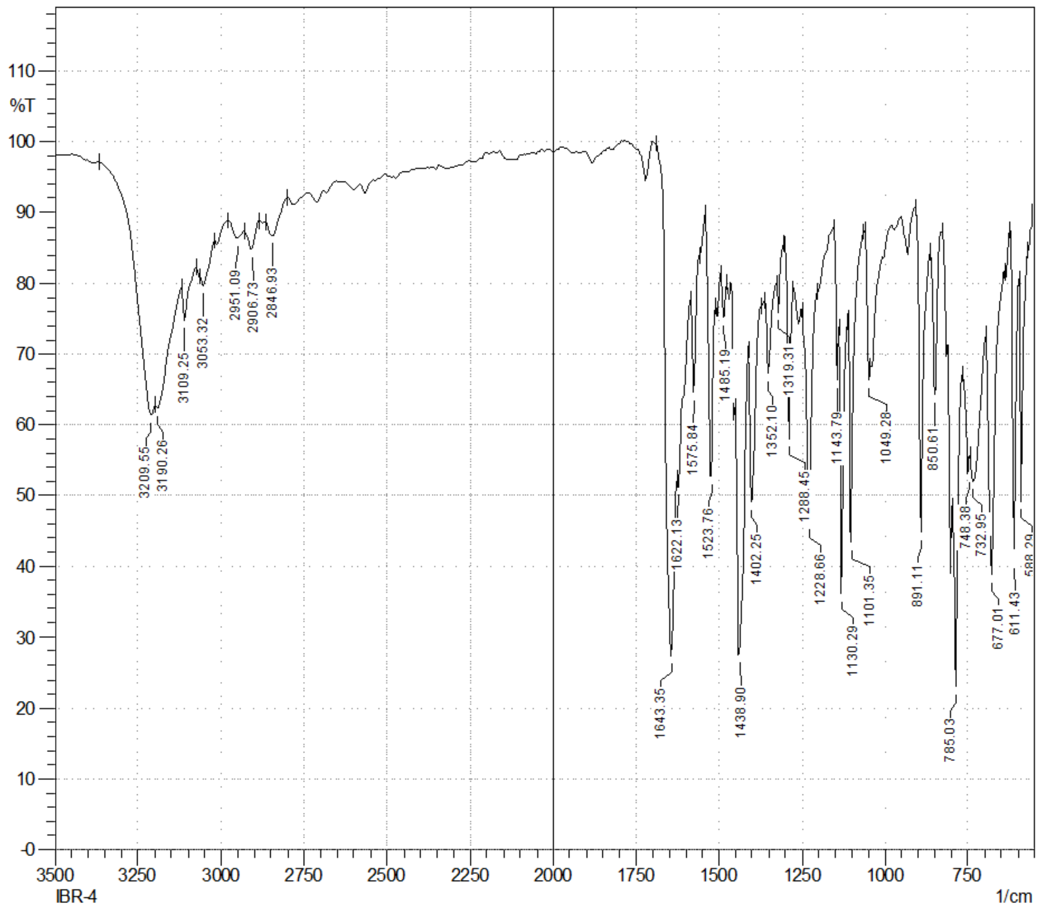

Figure S2. ^{1}H NMR spectrum of compound **1**

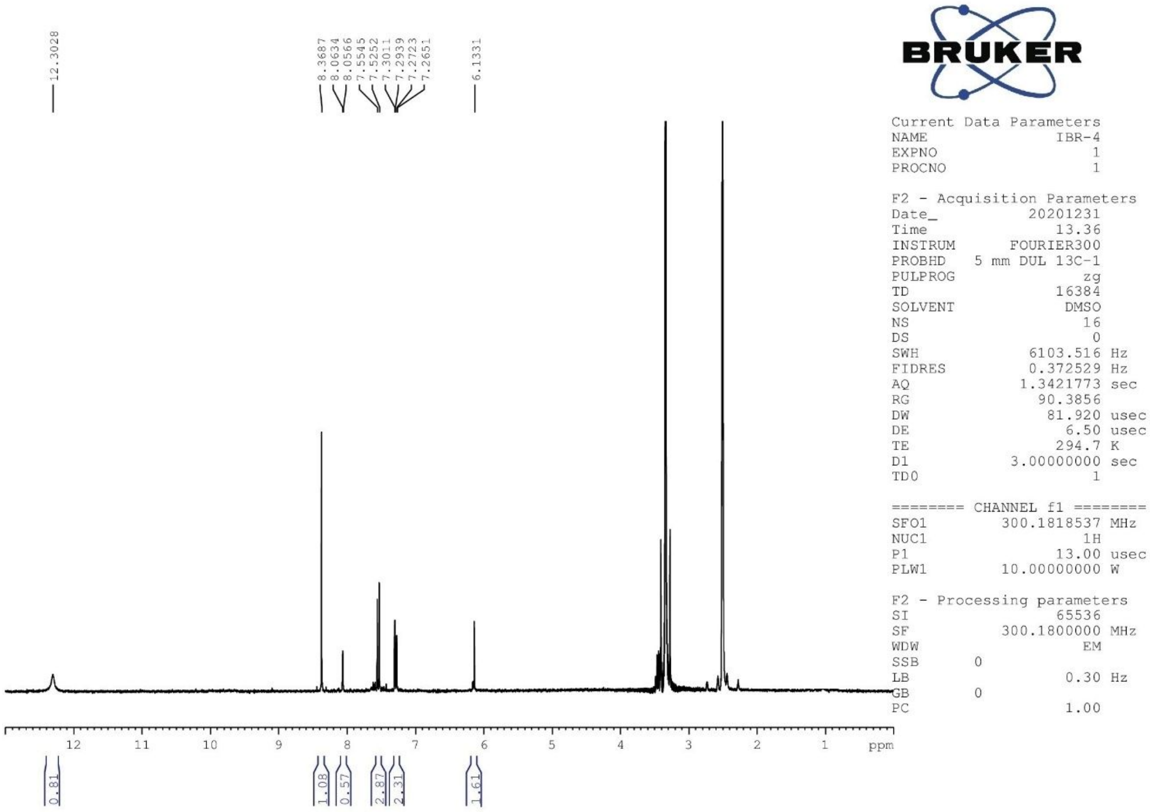

Figure S3. HRMS spectrum of compound **1**

Formula Predictor Report - IBR-4_34.lcd | Page 1 of 1

Data File: C:\LabSolutions\Data\Analiz\mdaltintop\IBR-4_34.lcd

Elmt	Val.	Min	Max	Elmt	Val.	Min	Max	Elmt	Val.	Min	Max	Elmt	Val.	Min	Max	Use Adduct
H	1	5	40	O	2	0	3	S	2	0	0	Ru	2	0	0	H
C	4	5	35	F	1	0	0	Cl	1	0	1	Pd	2	0	0	
N	3	0	10	P	3	0	0	Br	1	0	0	I	3	0	0	

Error Margin (ppm):	5	DBE Range:	0.0 - 60.0	Electron Ions:	both
HC Ratio:	unlimited	Apply N Rule:	yes	Use MSn Info:	yes
Max Isotopes:	3	Isotope RI (%):	1.00	Isotope Res:	9000
MSn Iso RI (%):	10.00	MSn Logic Mode:	AND	Max Results:	150

Event#: 1 MS(E+) Ret. Time : 4.400 Scan# : 661

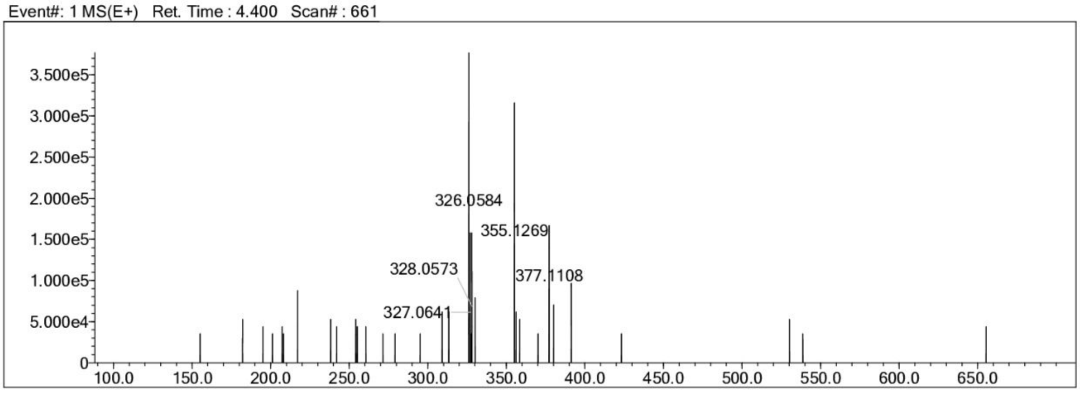

Measured region for 326.0584 m/z

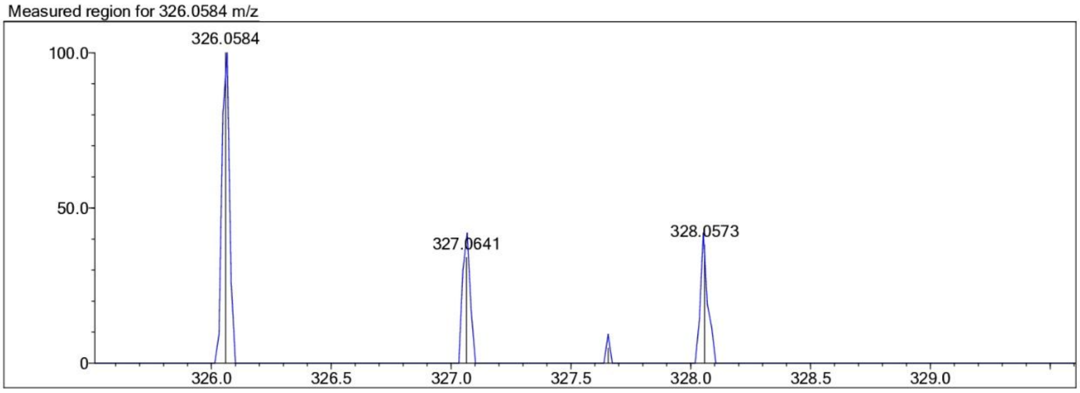

C18 H12 N O3 Cl [M+H]+ : Predicted region for 326.0578 m/z

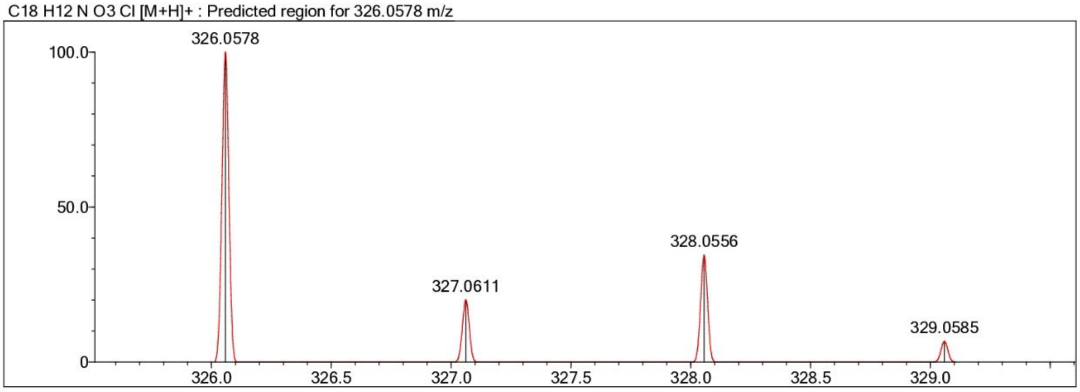

Rank	Score	Formula (M)	Ion	Meas. m/z	Pred. m/z	Df. (mDa)	Df. (ppm)	Iso	DBE
1	43.65	C18 H12 N O3 Cl	[M+H]+	326.0584	326.0578	0.6	1.84	44.59	13.0

Figure S4. IR spectrum of compound **2**

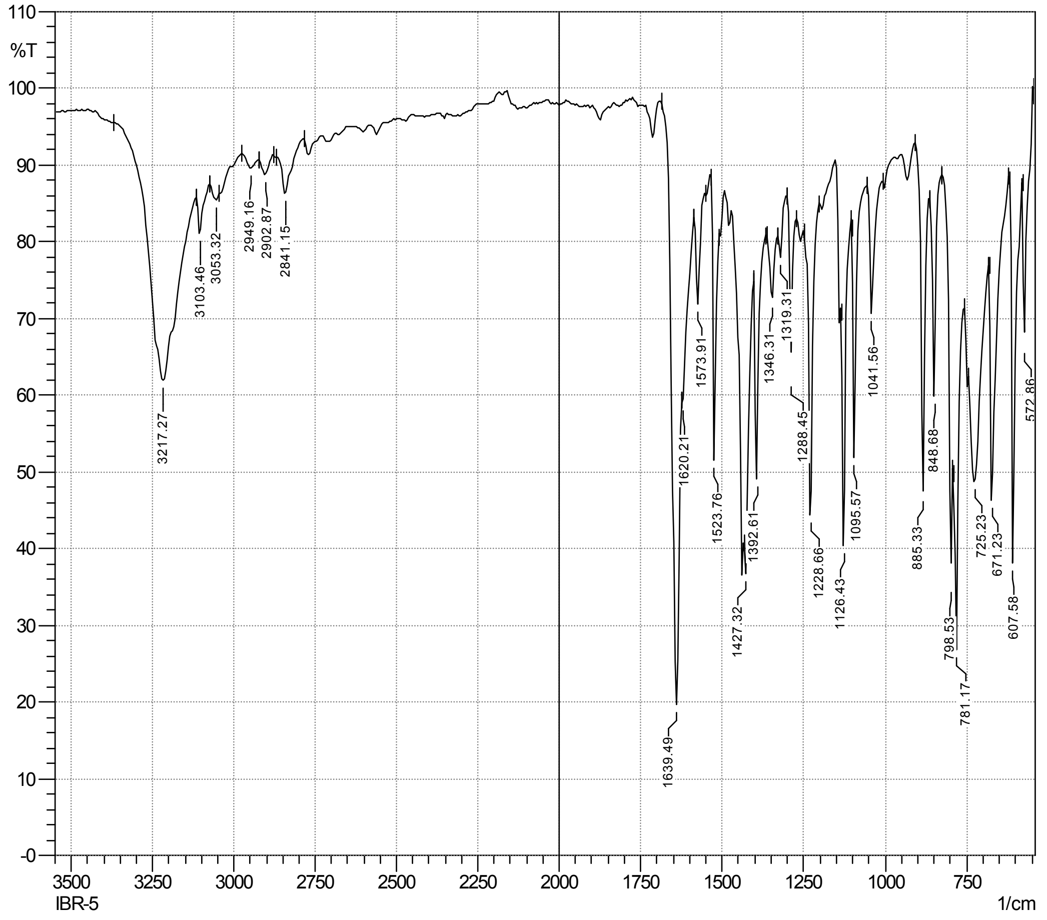

Figure S5. ^{1}H NMR spectrum of compound **2**

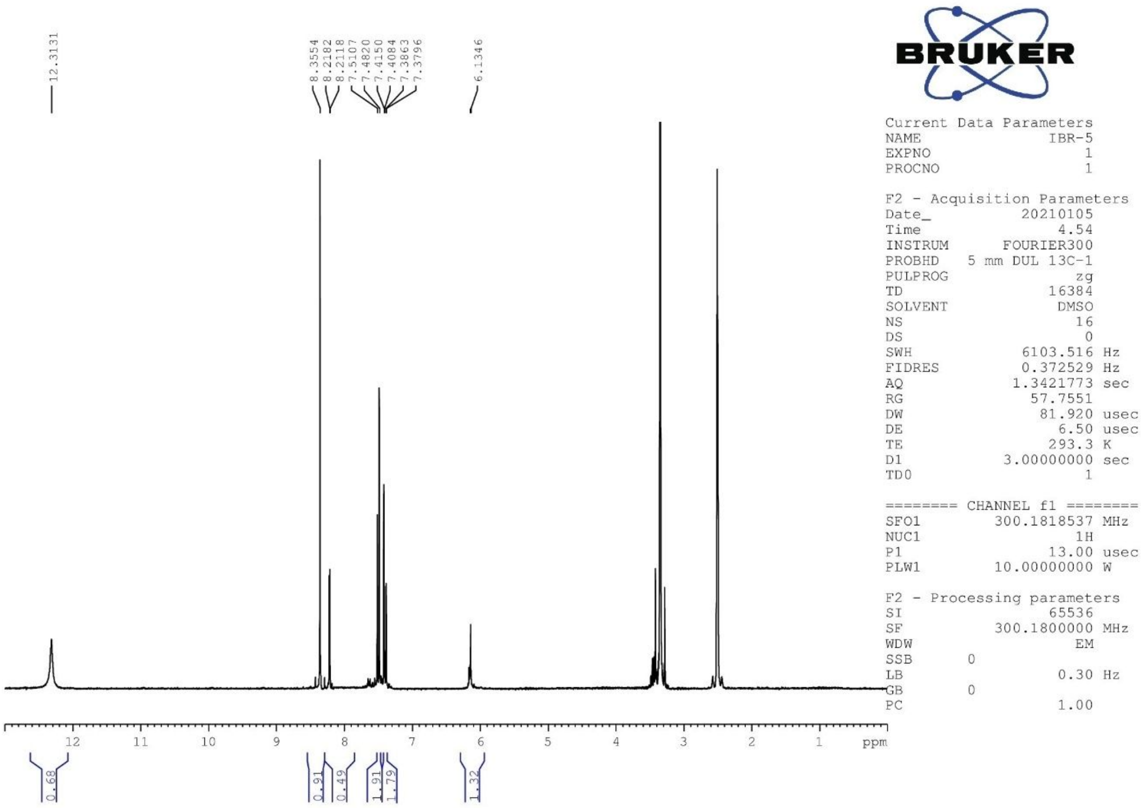

Figure S6. HRMS spectrum of compound **2**

Formula Predictor Report - IBR-5_35.lcd

Page 1 of 1

Data File: C:\LabSolutions\Data\Analiz\mdaltintop\IBR-5_35.lcd

Elmt	Val.	Min	Max	Elmt	Val.	Min	Max	Elmt	Val.	Min	Max	Elmt	Val.	Min	Max	Use Adduct
H	1	5	40	O	2	0	3	S	2	0	0	Ru	2	0	0	H
C	4	5	35	F	1	0	0	Cl	1	0	0	Pd	2	0	0	
N	3	0	5	P	3	0	0	Br	1	0	1	I	3	0	0	

Error Margin (ppm):	5	DBE Range:	0.0 - 60.0	Electron Ions:	both
HC Ratio:	unlimited	Apply N Rule:	yes	Use MSn Info:	yes
Max Isotopes:	3	Isotope RI (%):	1.00	Isotope Res:	9000
MSn Iso RI (%):	10.00	MSn Logic Mode:	AND	Max Results:	150

Event#: 1 MS(E+) Ret. Time : 4.560 Scan# : 685

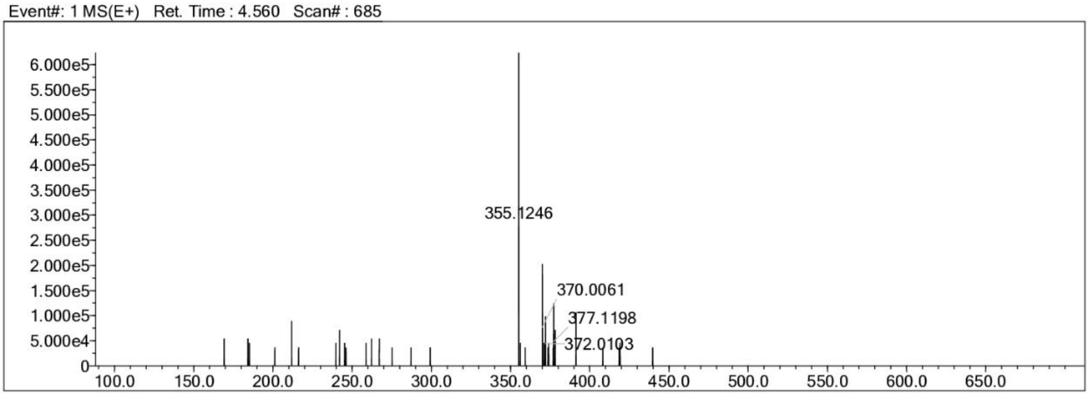

Measured region for 370.0061 m/z

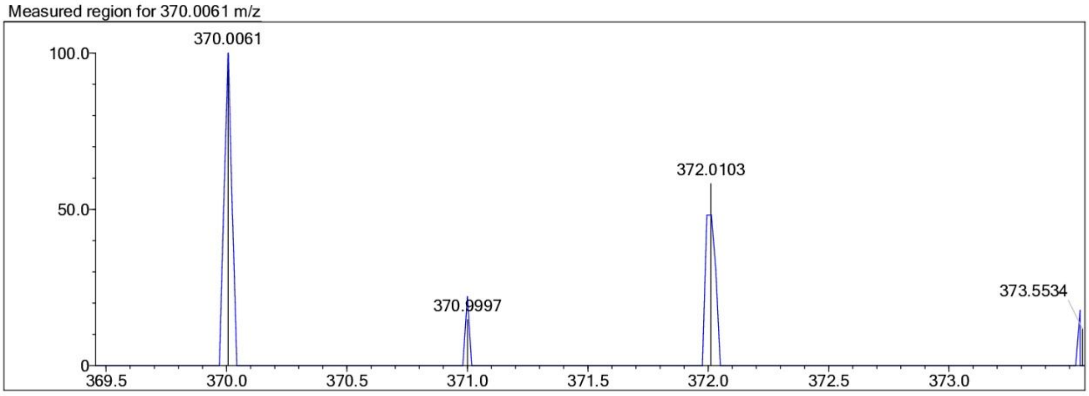

C18 H12 N O3 Br [M+H]+ : Predicted region for 370.0073 m/z

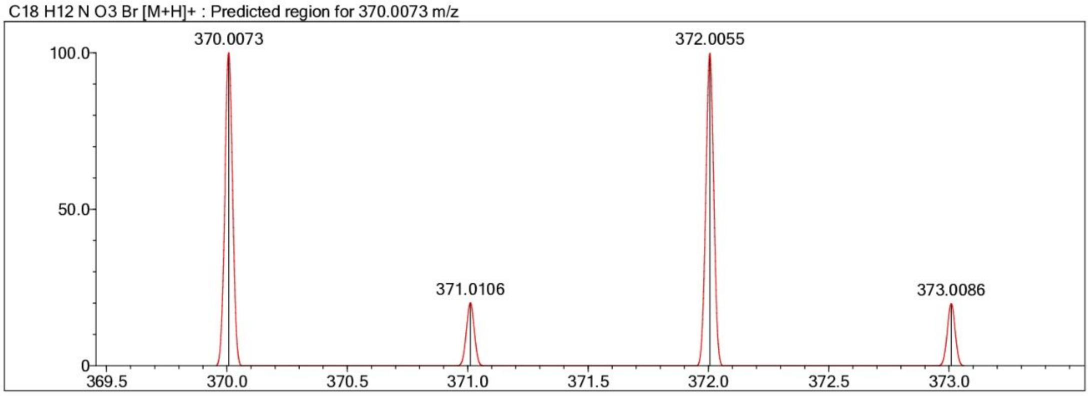

Rank	Score	Formula (M)	Ion	Meas. m/z	Pred. m/z	Df. (mDa)	Df. (ppm)	Iso	DBE
1	20.85	C18 H12 N O3 Br	[M+H]+	370.0061	370.0073	-1.2	-3.24	22.09	13.0

Figure S7. IR spectrum of compound **3**

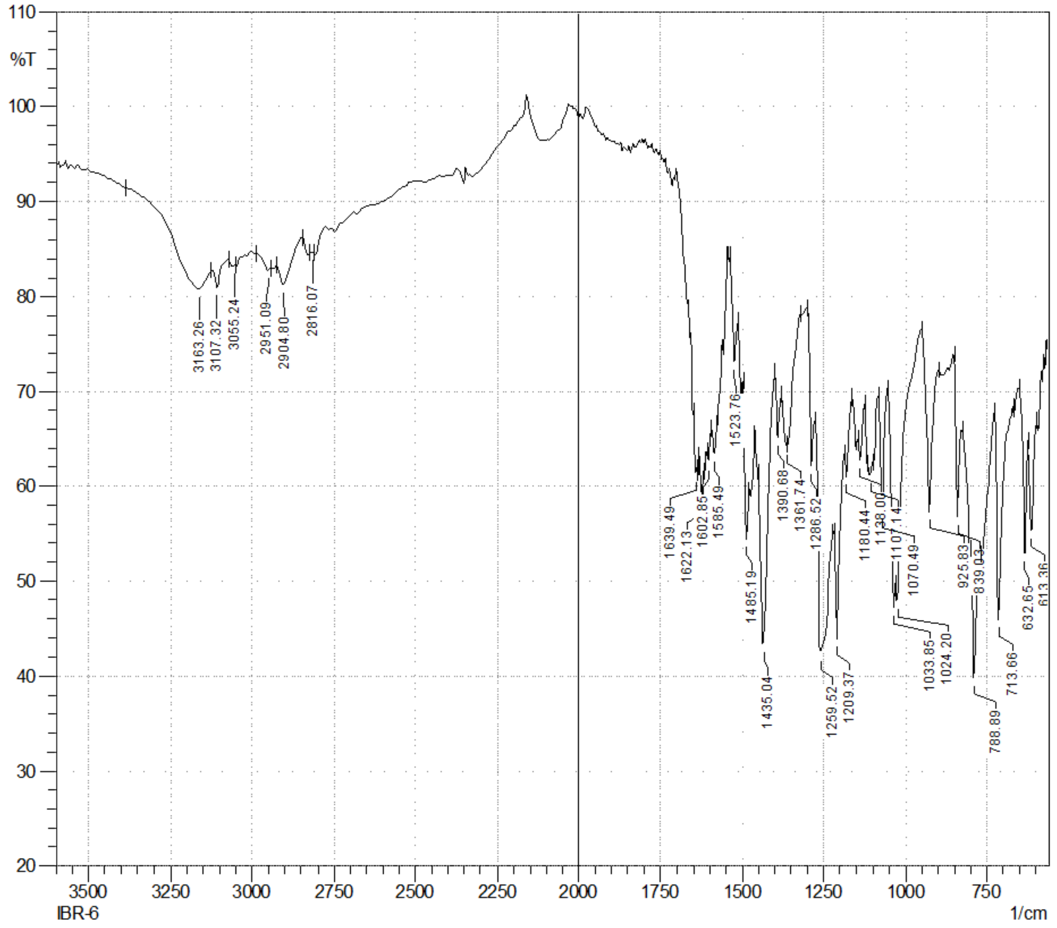

Figure S8. ^{1}H NMR spectrum of compound **3**

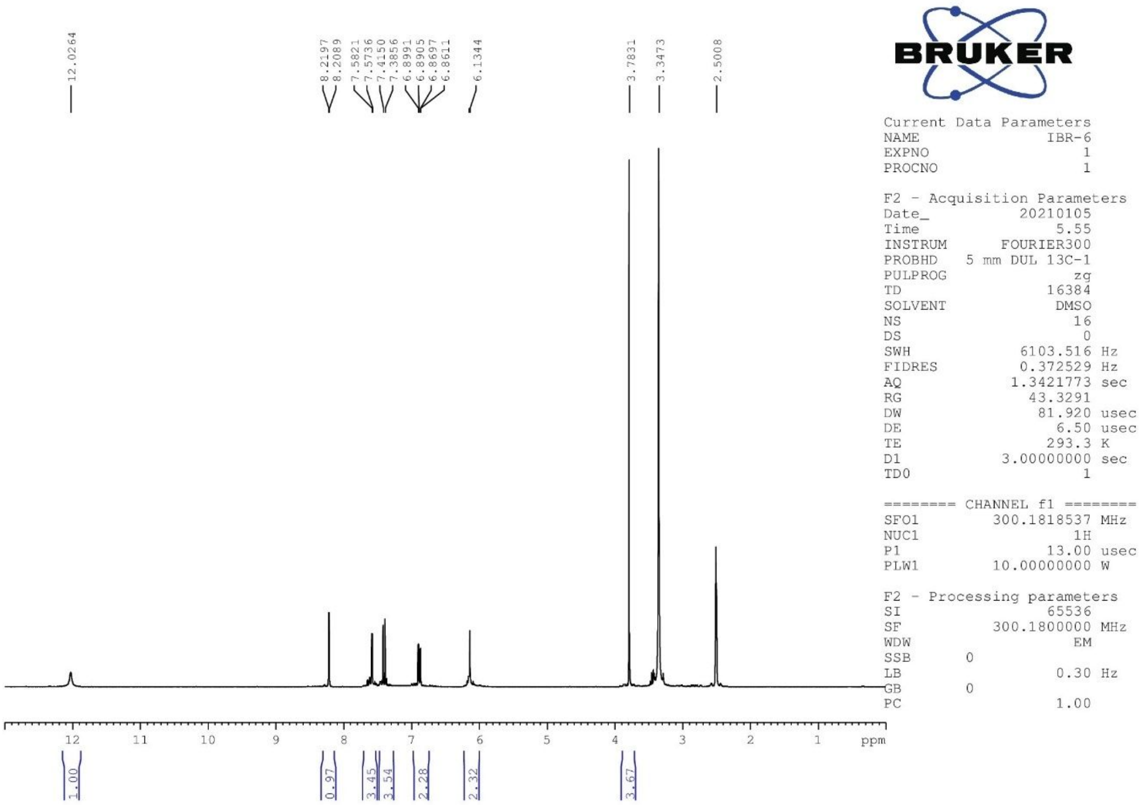

Figure S9. HRMS spectrum of compound **3**

Formula Predictor Report - IBR-6_36.lcd   Page 1 of 1

Data File: C:\LabSolutions\Data\Analiz\mdaltintop\IBR-6_36.lcd

Elmt	Val.	Min	Max	Elmt	Val.	Min	Max	Elmt	Val.	Min	Max	Elmt	Val.	Min	Max	Use Adduct
H	1	5	40	O	2	1	4	S	2	0	0	Ru	2	0	0	H
C	4	5	35	F	1	0	0	Cl	1	0	0	Pd	2	0	0	
N	3	0	5	P	3	0	0	Br	1	0	0	I	3	0	0	

Error Margin (ppm):	5	DBE Range:	0.0 - 60.0	Electron Ions:	both
HC Ratio:	unlimited	Apply N Rule:	yes	Use MSn Info:	yes
Max Isotopes:	3	Isotope RI (%):	1.00	Isotope Res:	9000
MSn Iso RI (%):	10.00	MSn Logic Mode:	AND	Max Results:	150

Event#: 1 MS(E+)   Ret. Time : 3.373   Scan# : 507

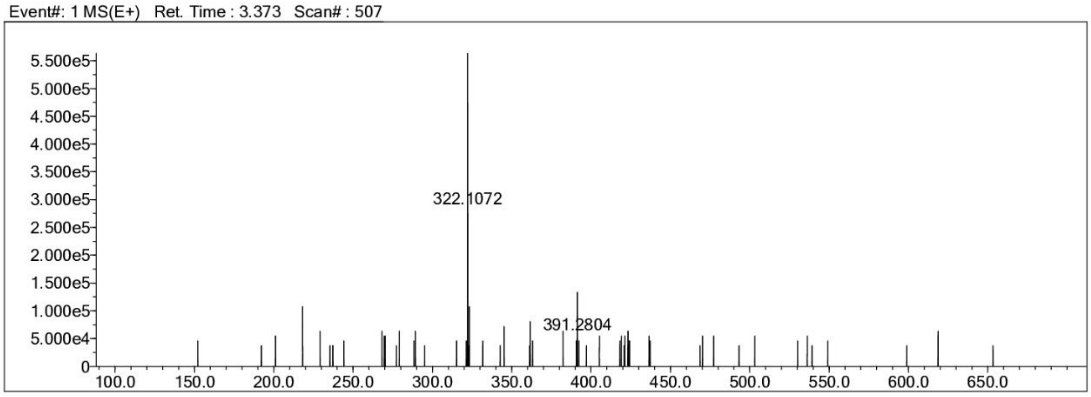

Measured region for 322.1072 m/z

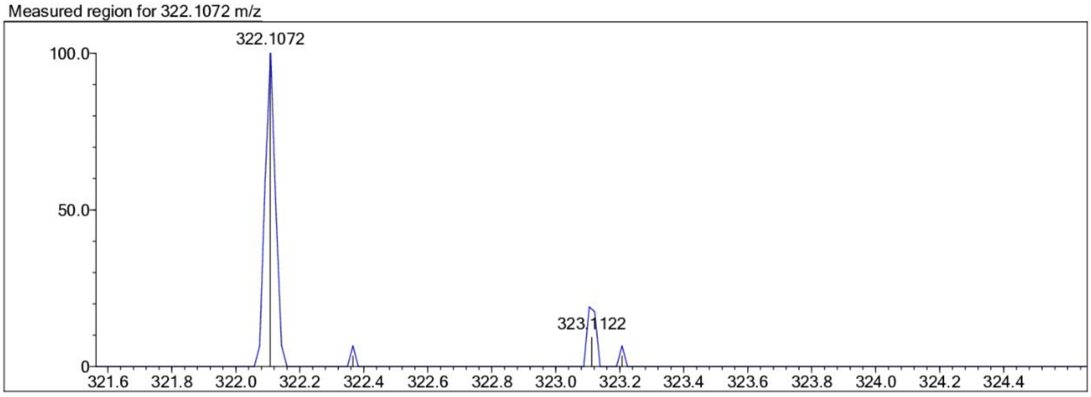

C19 H15 N O4 [M+H]+ : Predicted region for 322.1074 m/z

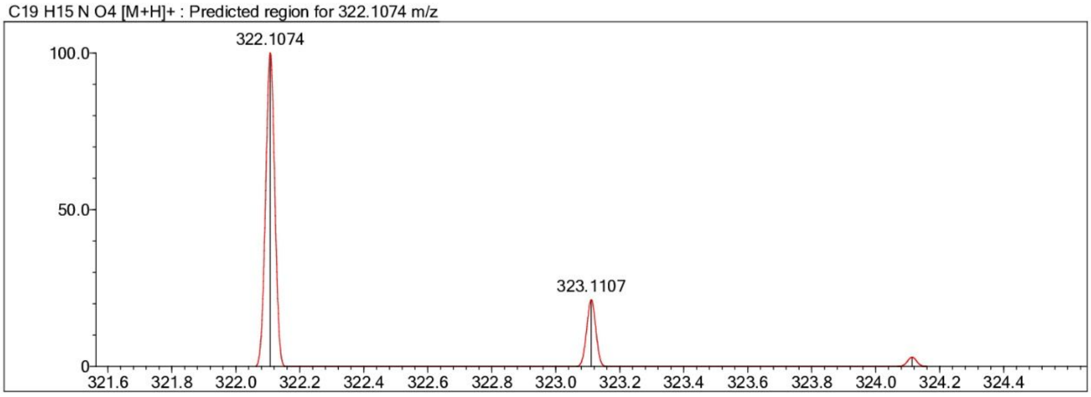

Rank	Score	Formula (M)	Ion	Meas. m/z	Pred. m/z	Df. (mDa)	Df. (ppm)	Iso	DBE
1	88.81	C19 H15 N O4	[M+H]+	322.1072	322.1074	-0.2	-0.62	88.81	13.0

Figure S10. IR spectrum of compound **4**

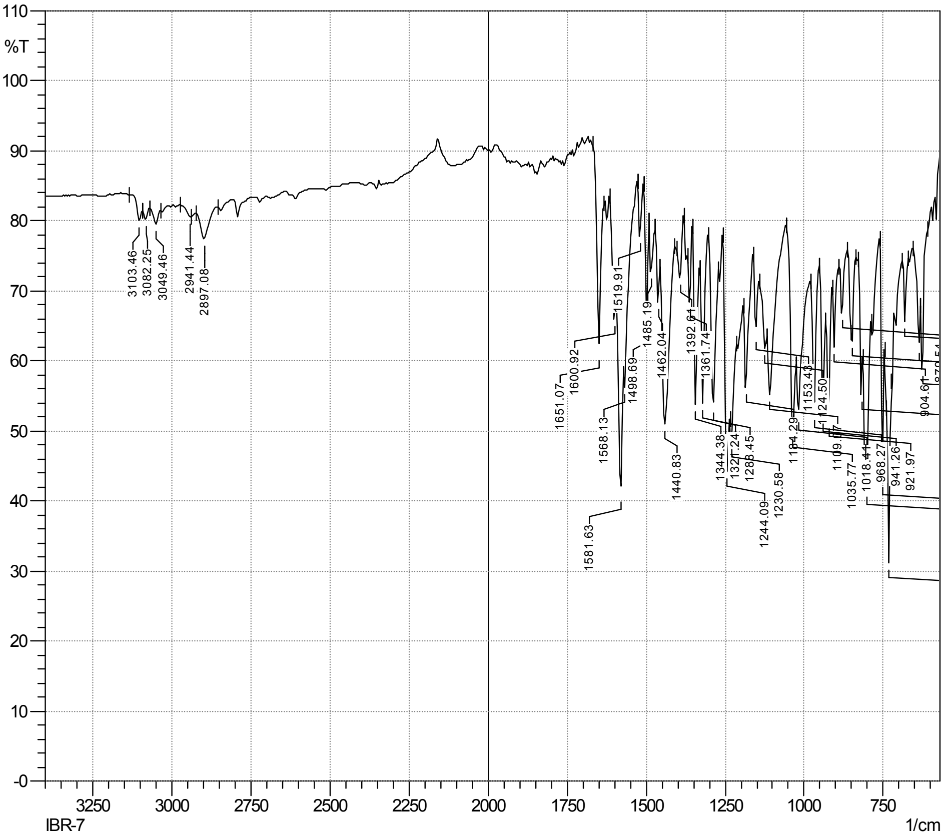

Figure S11. ^{1}H NMR spectrum of compound **4**

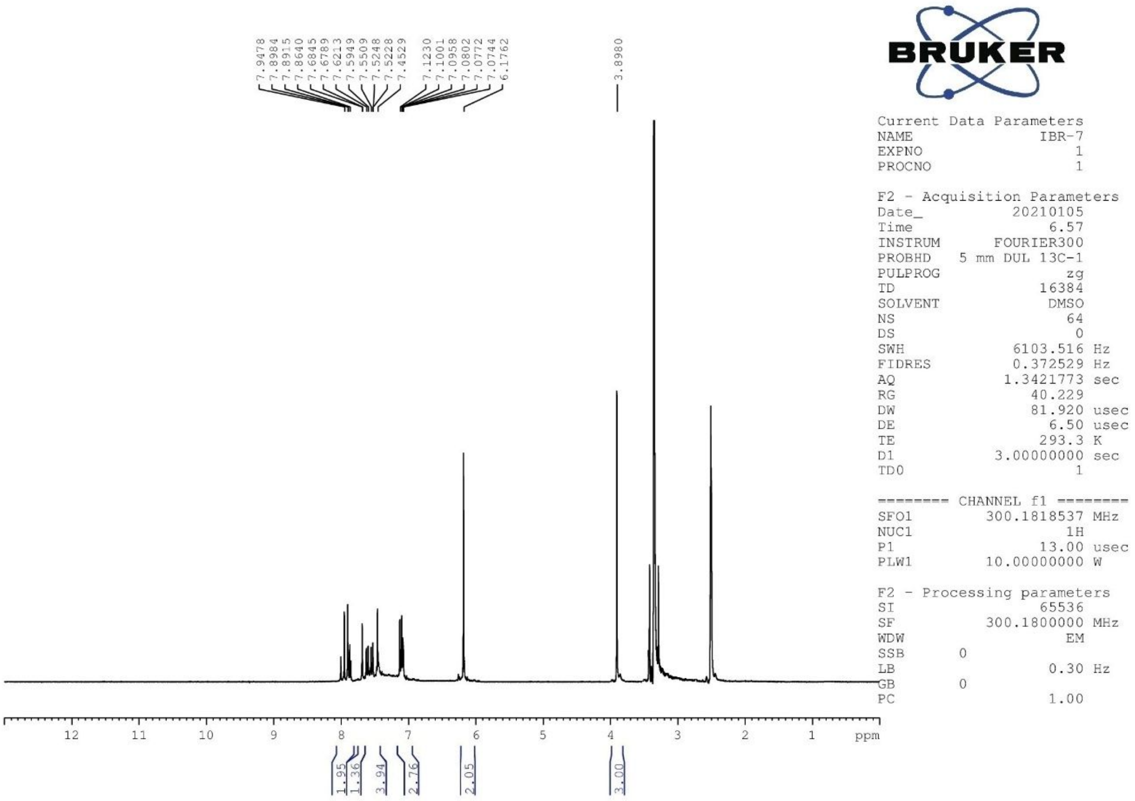

Figure S12. HRMS spectrum of compound **4**

Formula Predictor Report - IBR-7_37.lcd

Data File: C:\LabSolutions\Data\Analiz\mdaltintop\IBR-7_37.lcd

Elmt	Val.	Min	Max	Elmt	Val.	Min	Max	Elmt	Val.	Min	Max	Elmt	Val.	Min	Max	Use Adduct
H	1	5	40	O	2	1	4	S	2	0	0	Ru	2	0	0	H
C	4	5	35	F	1	0	0	Cl	1	0	0	Pd	2	0	0	
N	3	0	5	P	3	0	0	Br	1	0	0	I	3	0	0	

Error Margin (ppm):	5	DBE Range:	0.0 - 60.0	Electron Ions:	both
HC Ratio:	unlimited	Apply N Rule:	yes	Use MSn Info:	yes
Max Isotopes:	3	Isotope RI (%):	1.00	Isotope Res:	9000
MSn Iso RI (%):	10.00	MSn Logic Mode:	AND	Max Results:	150

Event#: 1 MS(E+) Ret. Time : 6.747 Scan# : 1013

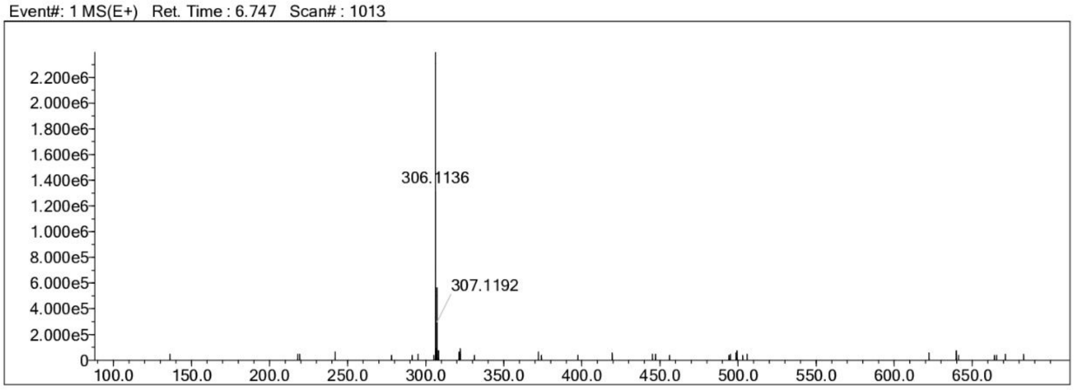

Measured region for 306.1136 m/z

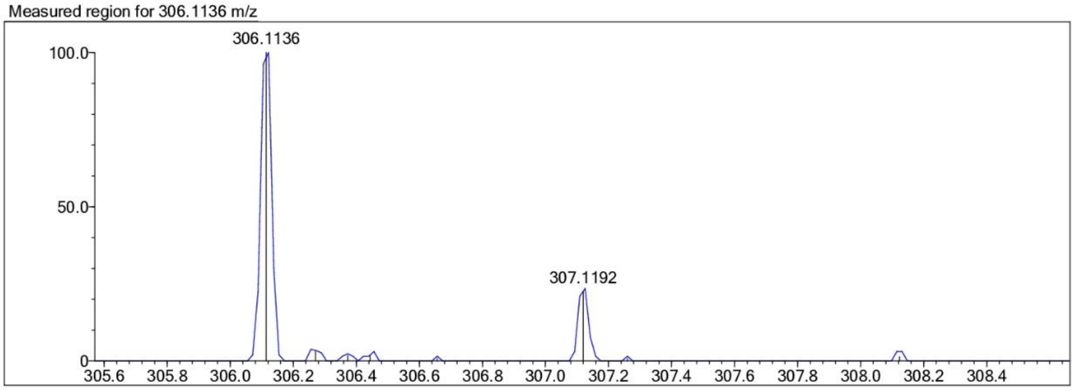

C19 H15 N O3 [M+H]+ : Predicted region for 306.1125 m/z

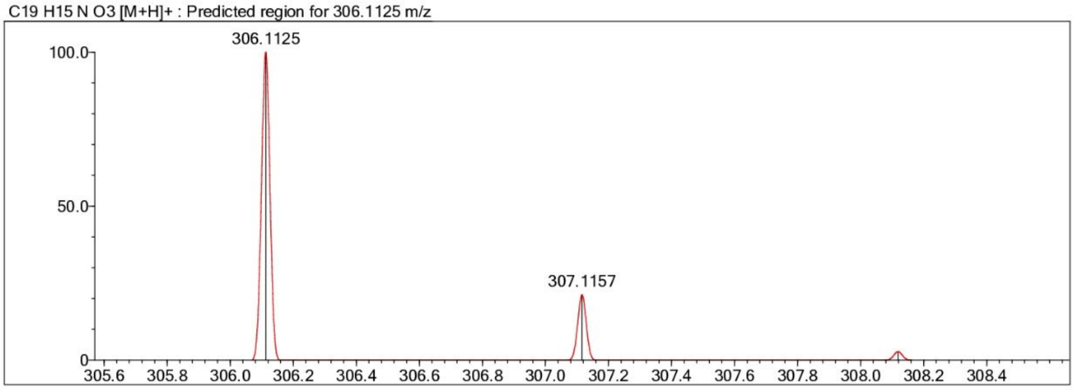

Rank	Score	Formula (M)	Ion	Meas. m/z	Pred. m/z	Df. (mDa)	Df. (ppm)	Iso	DBE
1	80.23	C19 H15 N O3	[M+H]+	306.1136	306.1125	1.1	3.59	85.79	13.0

Figure S13. IR spectrum of compound **5**

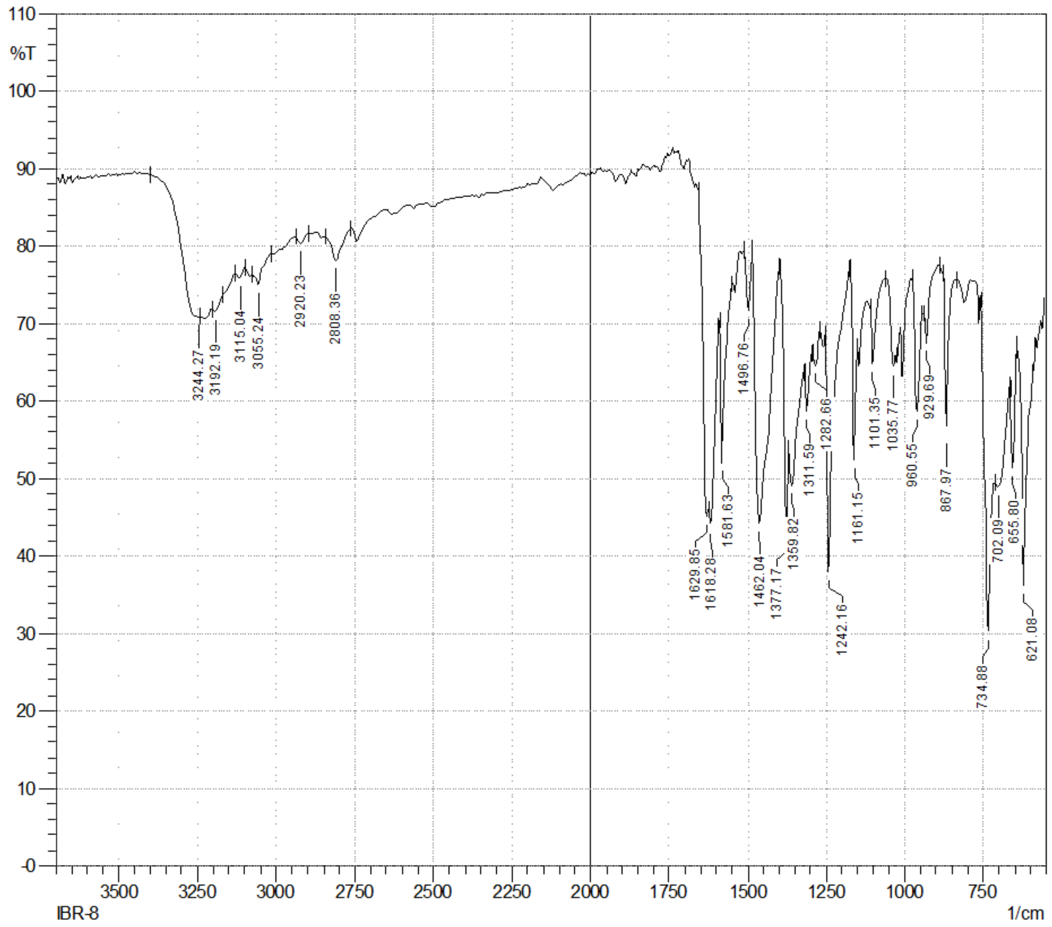

Figure S14. ^{1}H NMR spectrum of compound **5**

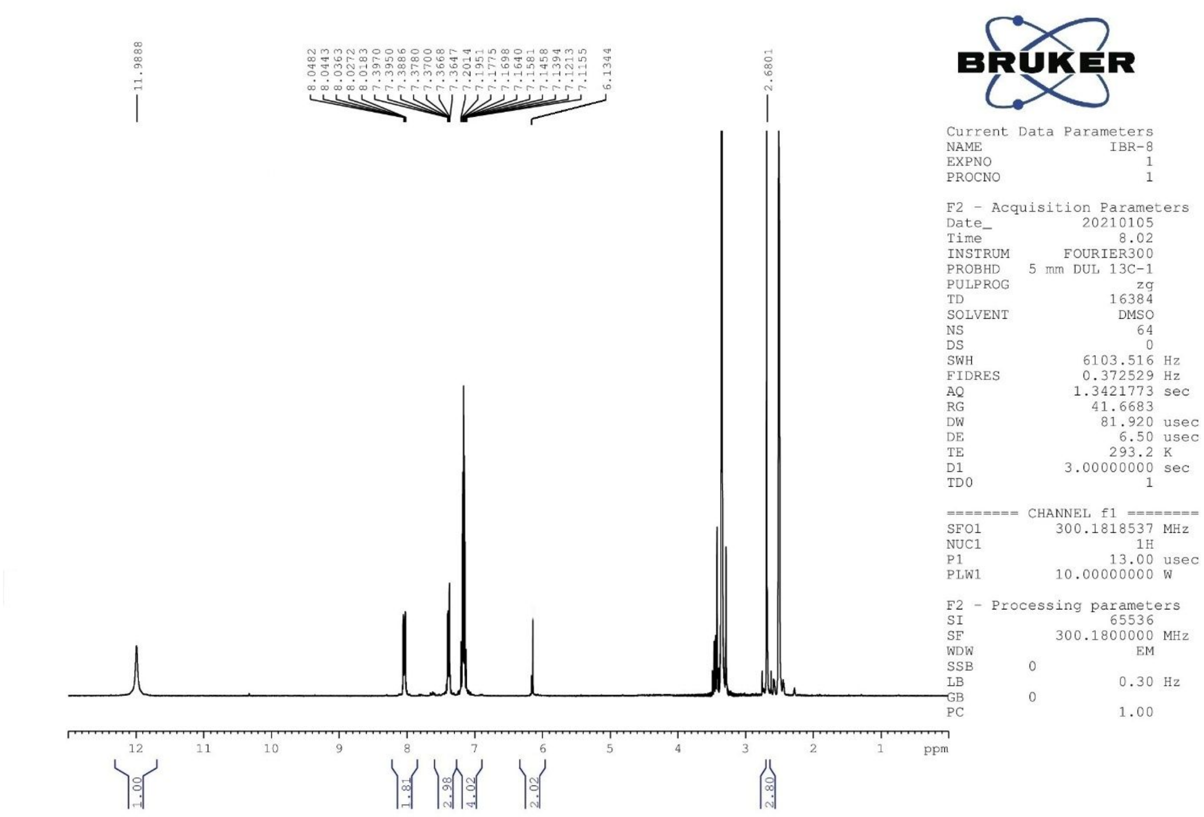

Figure S15. HRMS spectrum of compound **5**

Data File: C:\LabSolutions\Data\Analiz\mdaltintop\IBR-8_38.lcd

Elmt	Val.	Min	Max	Elmt	Val.	Min	Max	Elmt	Val.	Min	Max	Elmt	Val.	Min	Max	Use Adduct
H	1	5	40	O	2	1	4	S	2	0	0	Ru	2	0	0	H
C	4	5	35	F	1	0	0	Cl	1	0	0	Pd	2	0	0	
N	3	0	5	P	3	0	0	Br	1	0	0	I	3	0	0	

Error Margin (ppm):	5	DBE Range:	0.0 - 60.0	Electron Ions:	both
HC Ratio:	unlimited	Apply N Rule:	yes	Use MSn Info:	yes
Max Isotopes:	3	Isotope RI (%):	1.00	Isotope Res:	9000
MSn Iso RI (%):	10.00	MSn Logic Mode:	AND	Max Results:	150

Event#: 1 MS(E+) Ret. Time : 3.507 Scan# : 527

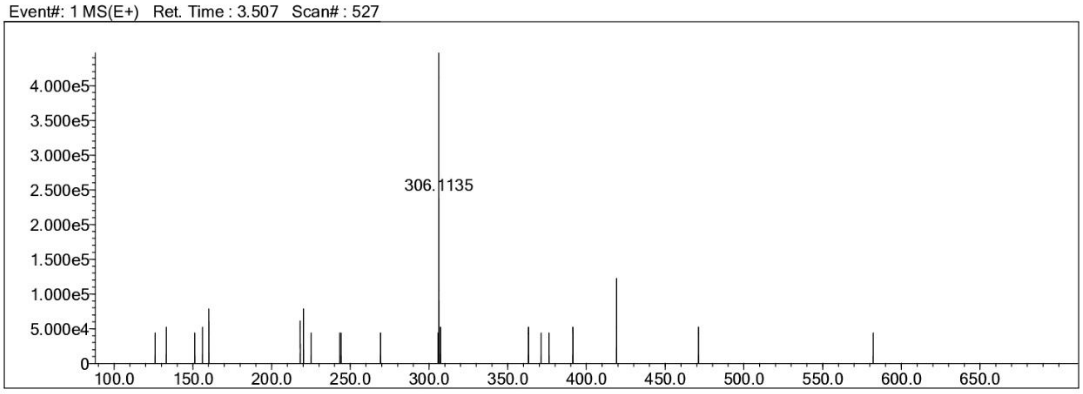

Measured region for 306.1135 m/z

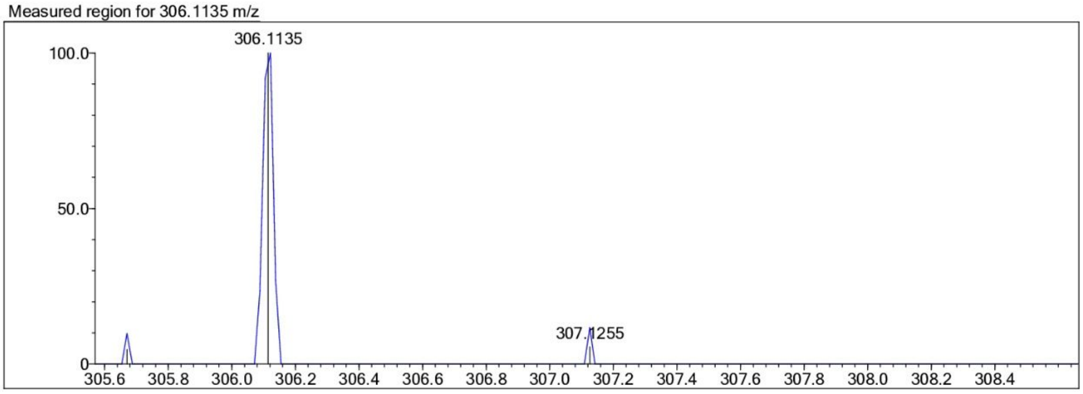

C19 H15 N O3 [M+H]+ : Predicted region for 306.1125 m/z

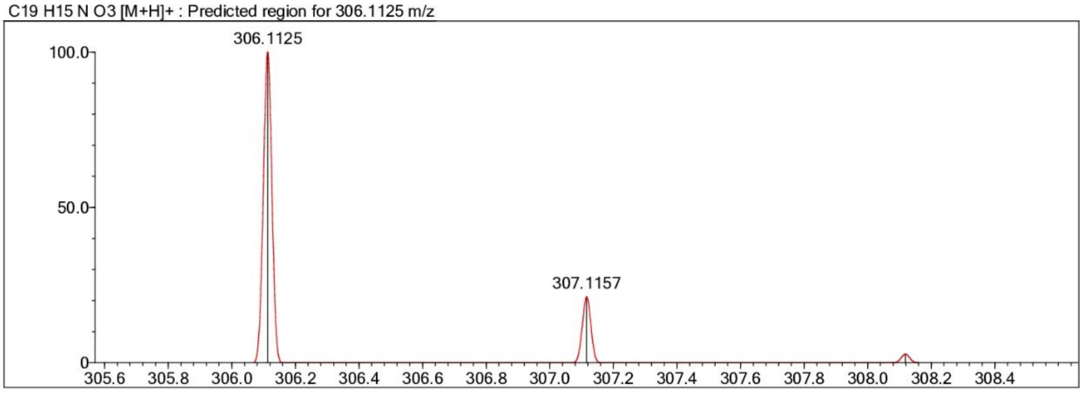

Rank	Score	Formula (M)	Ion	Meas. m/z	Pred. m/z	Df. (mDa)	Df. (ppm)	Iso	DBE
1	22.70	C19 H15 N O3	[M+H]+	306.1135	306.1125	1.0	3.27	24.06	13.0

Figure S16. IR spectrum of compound **6**

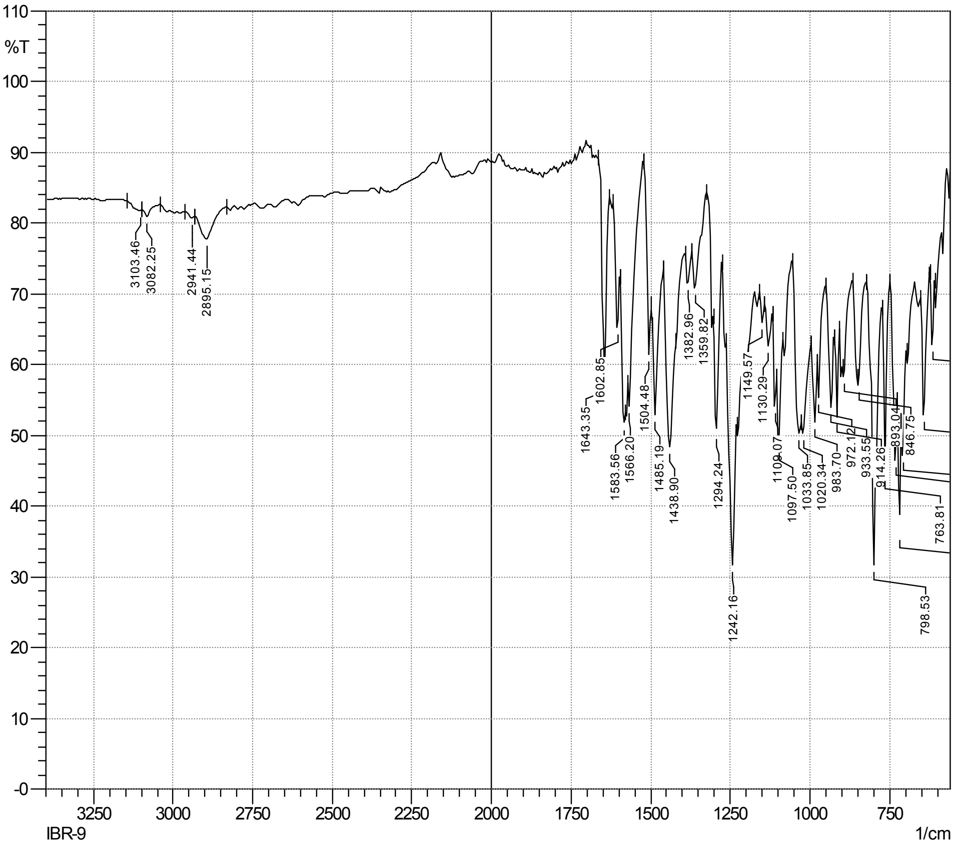

Figure S17. ^{1}H NMR spectrum of compound **6**

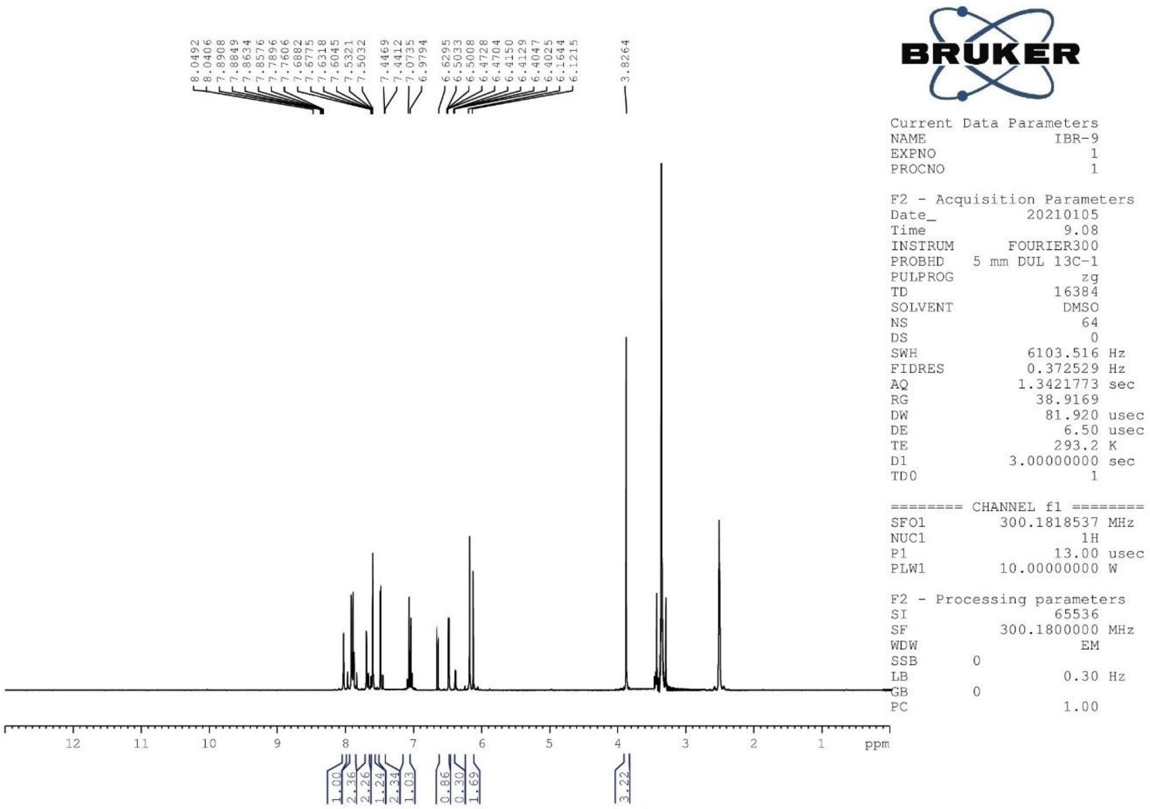

Figure S18. HRMS spectrum of compound **6**

Formula Predictor Report - IBR-9_39.lcd　　Page 1 of 1

Data File: C:\LabSolutions\Data\Analiz\mdaltintop\IBR-9_39.lcd

Elmt	Val.	Min	Max	Elmt	Val.	Min	Max	Elmt	Val.	Min	Max	Elmt	Val.	Min	Max	Use Adduct
H	1	5	40	O	2	1	4	S	2	0	0	Ru	2	0	0	H
C	4	5	35	F	1	0	0	Cl	1	0	0	Pd	2	0	0	
N	3	0	5	P	3	0	0	Br	1	0	0	I	3	0	0	

Error Margin (ppm):	5	DBE Range:	0.0 - 60.0	Electron Ions:	both
HC Ratio:	unlimited	Apply N Rule:	yes	Use MSn Info:	yes
Max Isotopes:	3	Isotope RI (%):	1.00	Isotope Res:	9000
MSn Iso RI (%):	10.00	MSn Logic Mode:	AND	Max Results:	150

Event#: 1 MS(E+)　Ret. Time : 5.093　Scan# : 765

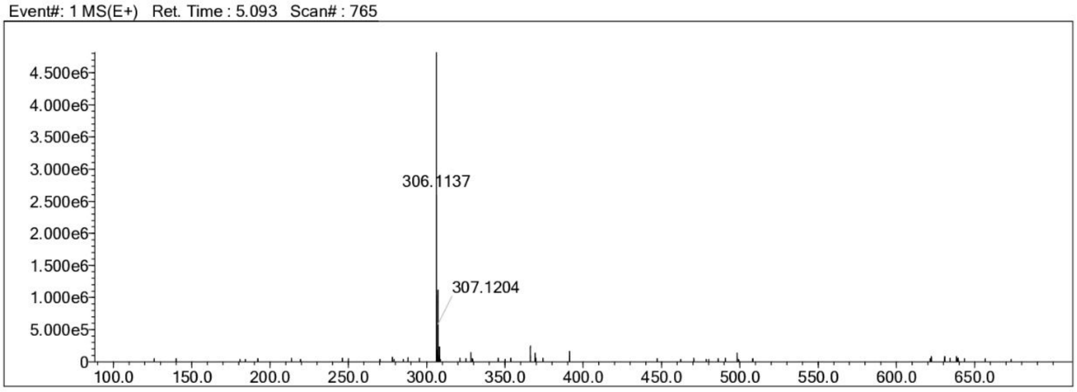

Measured region for 306.1137 m/z

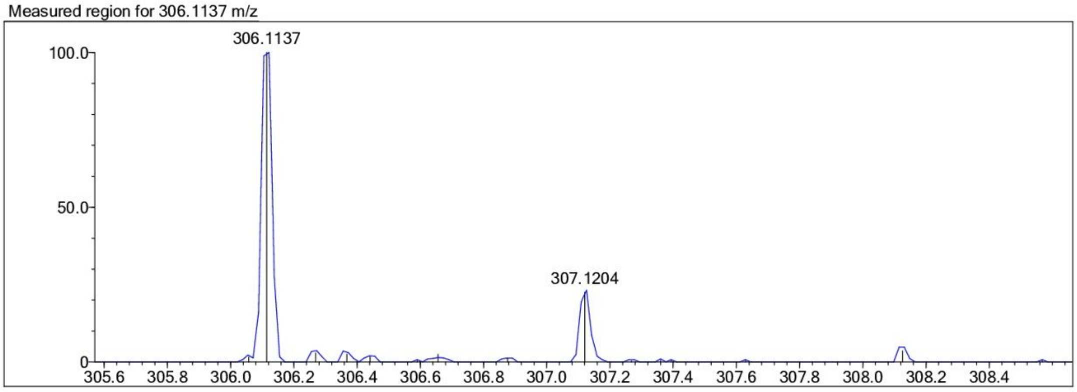

C19 H15 N O3 [M+H]+ : Predicted region for 306.1125 m/z

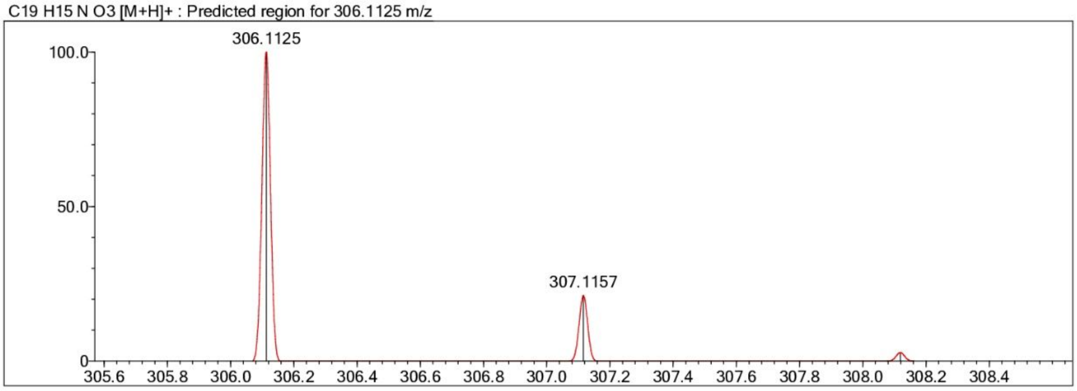

Rank	Score	Formula (M)	Ion	Meas. m/z	Pred. m/z	Df. (mDa)	Df. (ppm)	Iso	DBE
1	73.88	C19 H15 N O3	[M+H]+	306.1137	306.1125	1.2	3.92	79.70	13.0

Figure S19. IR spectrum of compound **7**

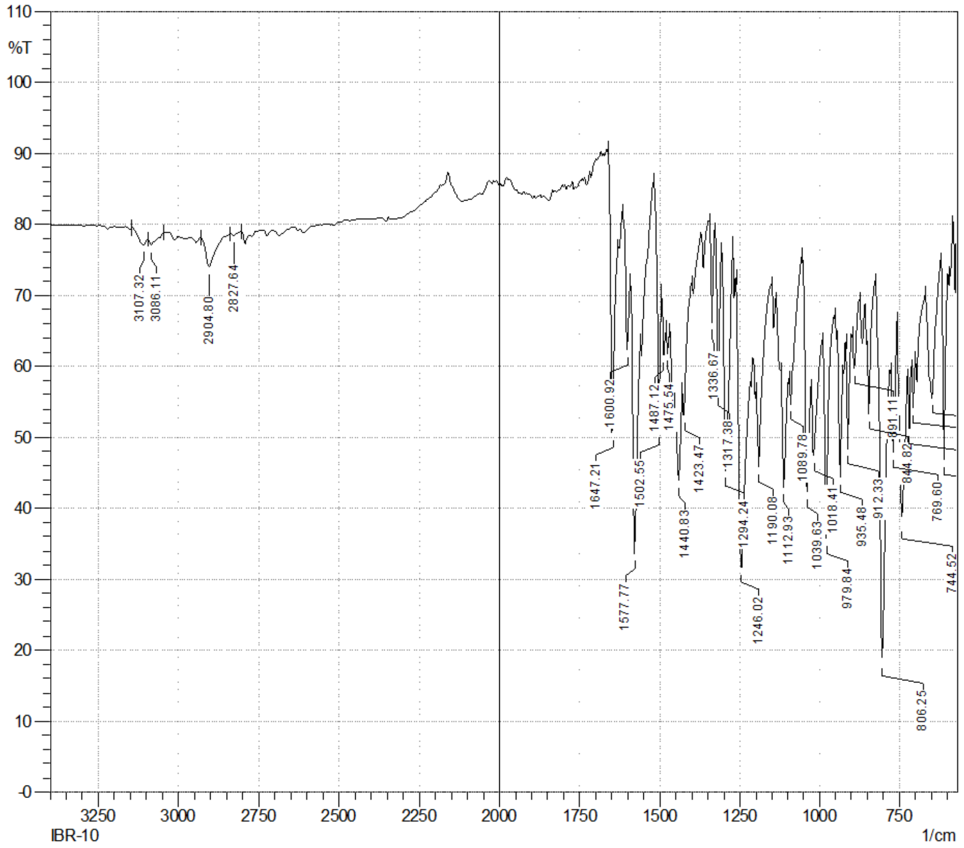

Figure S20. ^{1}H NMR spectrum of compound **7**

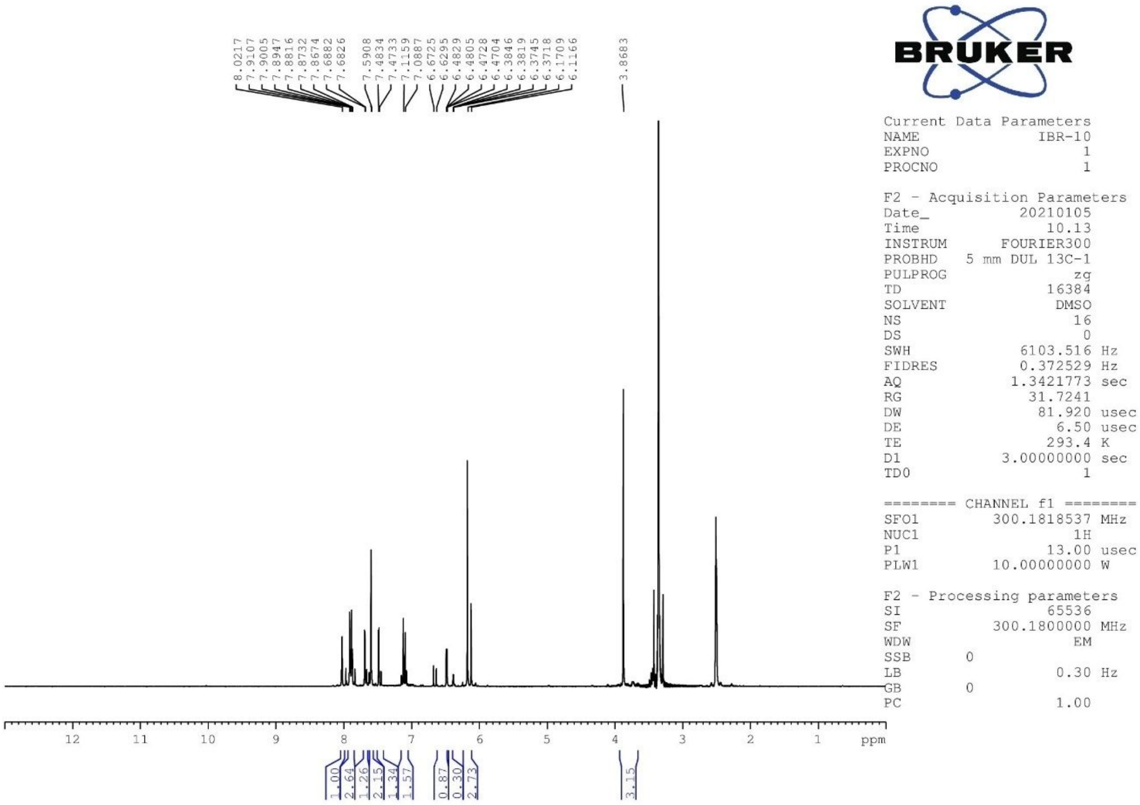

Figure S21. HRMS spectrum of compound **7**

Formula Predictor Report - IBR-10_40.lcd

Data File: C:\LabSolutions\Data\Analiz\mdaltintop\IBR-10_40.lcd

Elmt	Val.	Min	Max	Elmt	Val.	Min	Max	Elmt	Val.	Min	Max	Elmt	Val.	Min	Max	Use Adduct
H	1	5	40	O	2	1	4	S	2	0	0	Ru	2	0	0	H
C	4	5	35	F	1	0	0	Cl	1	0	0	Pd	2	0	0	
N	3	0	5	P	3	0	0	Br	1	0	0	I	3	0	0	

Error Margin (ppm):	5	DBE Range:	0.0 - 60.0	Electron Ions:	both
HC Ratio:	unlimited	Apply N Rule:	yes	Use MSn Info:	yes
Max Isotopes:	3	Isotope RI (%):	1.00	Isotope Res:	9000
MSn Iso RI (%):	10.00	MSn Logic Mode:	AND	Max Results:	150

Event#: 1 MS(E+) Ret. Time : 5.613 Scan# : 843

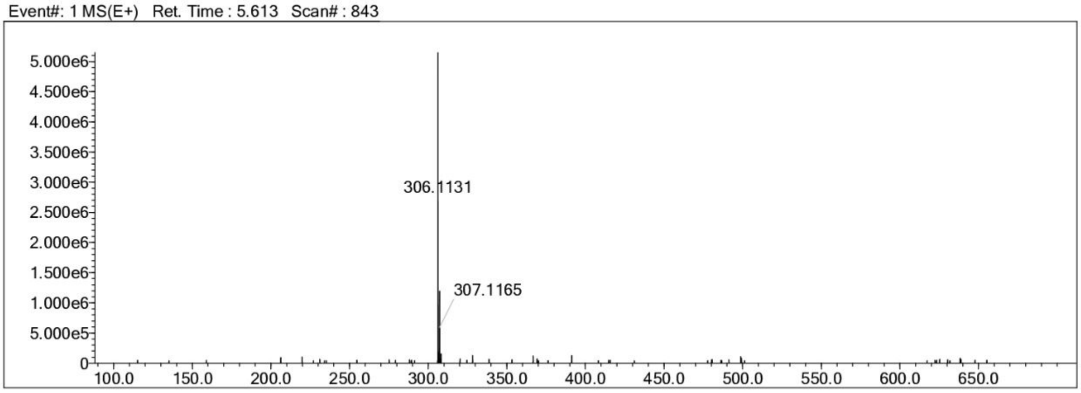

Measured region for 306.1131 m/z

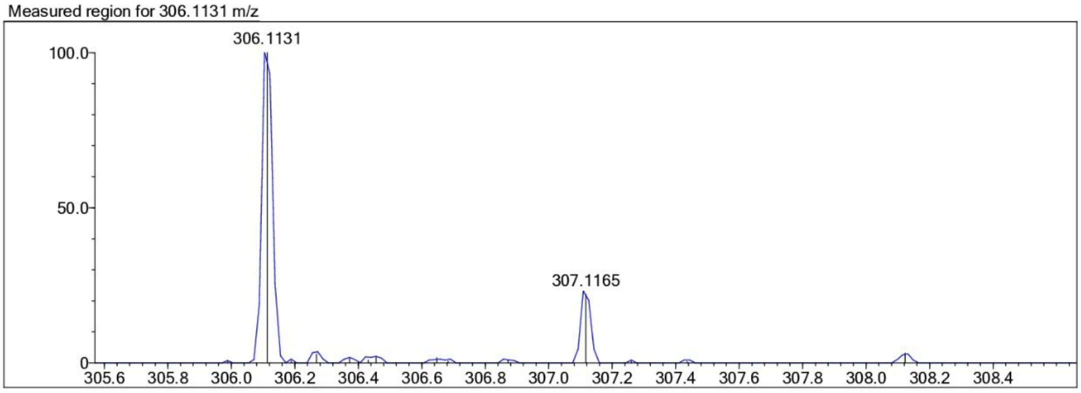

C19 H15 N O3 [M+H]+ : Predicted region for 306.1125 m/z

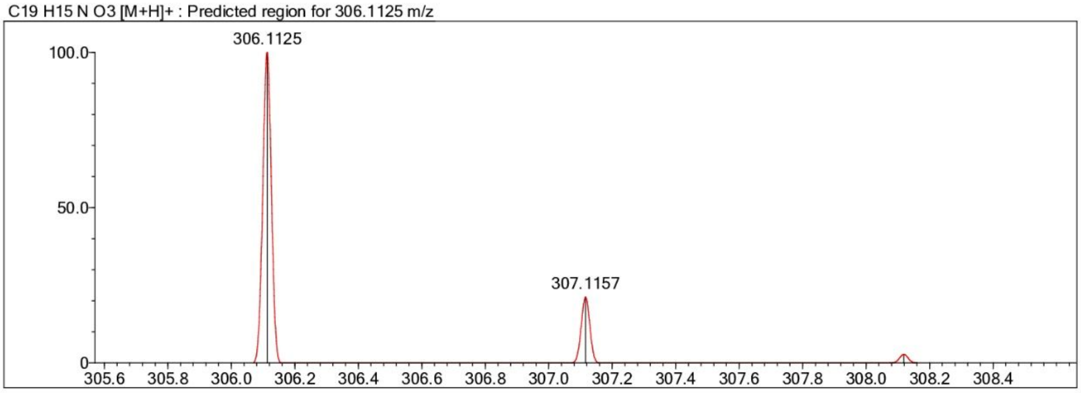

Rank	Score	Formula (M)	Ion	Meas. m/z	Pred. m/z	Df. (mDa)	Df. (ppm)	Iso	DBE
1	86.52	C19 H15 N O3	[M+H]+	306.1131	306.1125	0.6	1.96	88.65	13.0
